# Thermal stress impairs photophysiology and redox balance in the kelp *Lessonia spicata*

**DOI:** 10.3389/fpls.2025.1727961

**Published:** 2025-12-15

**Authors:** Camilo Navarrete, Macarena Troncoso, Céline Lavergne, Verónica Molina, Paula S. M. Celis-Plá

**Affiliations:** 1 Doctorado Interdisciplinario en Ciencias Ambientales, Facultad de Ciencias Naturales y Exactas, Universidad de Playa Ancha, Valparaíso, Chile; 2 Laboratorio de Investigación Ambiental Acuático (LACER)/HUB Ambiental UPLA, Universidad de Playa Ancha, Valparaíso, Chile; 3 Departamento de Ciencias y Geografía, Facultad de Ciencias Naturales y Exactas, Universidad de Playa Ancha, Valparaíso, Chile; 4 Departamento de Biología Marina i Oceanografía, Instituto de Ciencias del Mar, ICM-CSIC, Barcelona, Spain; 5 HUB AMBIENTAL UPLA, Universidad de Playa Ancha, Valparaíso, Chile; 6 Centro de Investigación Oceanográfica COPAS COASTAL, Universidad de Concepción, Concepción, Chile

**Keywords:** kelp physiology, photoprotection, reactive oxygen species, antioxidant capacity, phenolics compounds

## Abstract

Warming oceans are reshaping coastal ecosystems, yet the thermal sensitivity of many foundation macroalgae remains poorly constrained. Under SSP2-4.5, sea surface temperatures are expected to rise by ~2.7 °C by 2100, with unknown consequences for the canopy-forming kelp Lessonia spicata. We exposed thalli to control (15 °C) and warming (18 °C) conditions for 14 days to simulate near-future thermal scenarios, assessing photophysiology, oxidative stress and pigment composition. Warming caused marked declines in F_v_/F_m_ and ETR_max_ and increased NPQ_max_, signaling reduced photochemical efficiency and enhanced thermal energy dissipation. Simultaneously, pigment concentrations decreased, ROS and MDA accumulated, and antioxidant capacity dropped, indicating rapid disruption of cellular redox homeostasis. Our results demonstrate that even modest warming consistent with mid-century projections triggers immediate photophysiological impairment and oxidative stress in *L. spicata*. These mechanistic insights highlight potential vulnerability of kelp forests in central Chile as ocean temperatures continue to rise.

## Graphical abstract

**Figure:**
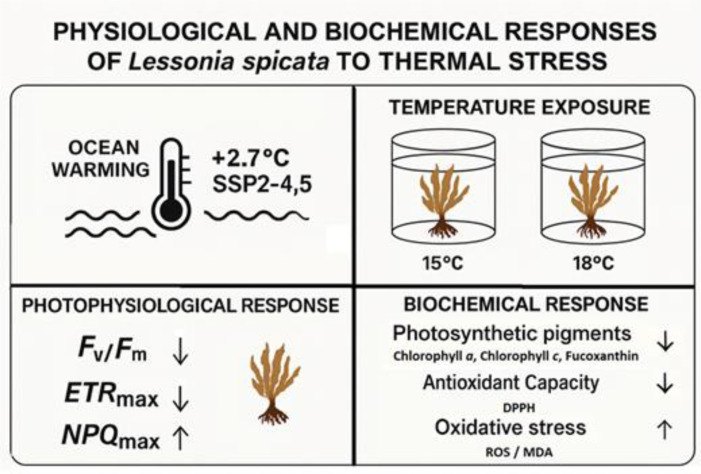

## Introduction

Canopy-forming brown macroalgae (kelp), such as *L. spicata* (Suhr) (resilient Phaeophyceae, Ochrophyta), are foundational species and among the most ecologically important primary producers in temperate coastal habitats worldwide. They play a critical role in structuring intertidal and subtidal habitats, contributing substantially to nutrient cycling, primary production, and providing essential habitat complexity (Buschmann et al., 2014; Gómez et al., 2016; Rosenfeld et al., 2019). Climatic change (CC) is one of the most urgent environmental threats of the 21^st^ century. Its impacts are particularly severe in marine ecosystems, where rising greenhouse gas emissions elevate sea surface temperatures (SST), leading to widespread ecological disruption (Dubash, 2020; IPCC, 2023). In temperate coastal regions, increasing SST imposes multiple stressors on kelp species, affecting key physiological processes such as photosynthesis, respiration, nutrient uptake, and life-cycle dynamics (Harley et al., 2006; Smale, 2020; Gundersen et al., 2021; Tregarot et al., 2024). These physiological disturbances can compromise not only individual performance but also the persistence of entire populations of primary producers over time. Moreover, warming is causing unprecedented shifts in species distributions, triggering biodiversity loss, habitat degradation, and reorganization of biological communities, and the emergence of novel species assemblages, which may alter ecosystem services in ways we are only beginning to understand (Wernberg et al., 2019; Duarte et al., 2022). Such distributional shifts have already been predicted for canopy-forming macroalgae under climate warming scenarios; similar patterns have been predicted for other canopy-forming species using ecological niche models under warming conditions (Carneiro et al., 2023).

Projections under SSP2–4.5 scenarios, estimate a rise of +2.7 °C in SST by 2100 (IPCC, 2023). While moderate, this warming still poses critical risks to macroalgae, particularly foundational species in temperate marine ecosystems, which may exceed their physiological thermal limits. This is consistent with findings in other cold-adapted macroalgae exhibiting reduced thermal tolerance when exposed to increased irradiance and temperature (Marambio and Bischof, 2021). When such thresholds are surpassed, abrupt changes in abundance and geographical range can occur, with cascading consequences for ecosystem structure and function (Smale, 2020). The loss of kelp forests, for example, has already been documented in several regions, including a 43% decline in Western Australia between 2011 and 2015 because of extreme marine heatwaves (Coleman and Wernberg, 2017).

Their productivity and resilience strongly depend on efficient photosynthetic and photoprotective mechanisms, which are sensitive to temperature and irradiance. Photochemical indicators such as the maximum quantum yield of Photosystem II (*F*_v_/*F*_m_), the maximum electron transport rate ETR_max_ and non-photochemical quenching NPQ are widely used to quantify photosynthetic performance and stress tolerance in kelps. *F*_v_/*F*_m_ is the primary measure of photosynthetic efficiency and is highly sensitive to stress; its decline indicates photoinhibition and cellular damage to the photosystem II reaction centers. ETR_max_ represents the maximum rate of light-driven electron flow through the thylakoid membranes, serving as an indicator of maximum photosynthetic capacity. Finally, NPQ quantifies the dissipation of excess light energy as heat, acting as a crucial photoprotective mechanism under stress conditions (Figueroa et al., 2019). Integrating these metrics provides a robust understanding of the physiological status of macroalgae.

Multiple studies have documented strong physiological and biochemical impacts of elevated temperature on brown macroalgae. The decline in the maximum quantum yield of PSII (F_v_/F_m_) is a consistent indicator of thermal damage. For instance, *Cladosiphon okamuranus*, *Nemacystus decipiens*, and *Turbinaria ornata* exhibited significant drops in F_v_/F_m_ when exposed to elevated temperatures (Fukumoto et al., 2019; Narrain et al., 2023; Bell et al., 2024). Observed seasonal variation in *F*_v_/*F*_m_ values has been documented in *L. spicata*, highlighting its ecophysiological plasticity and capacity for photoacclimation to predictable environmental shifts (Zúñiga et al., 2021; Celis-Plá et al., 2022). However, this acclimation capacity is finite, and vulnerability increases when physiological thresholds are exceeded by acute events. Reductions in the maximum electron transport rate (ETR_max_) have been observed in *Fucus serratus* under elevated temperature, indicating impaired capacity for electron flow a distribution ranges and energy production (Figueroa et al., 2019). Similarly, elevated temperature exposure in other brown algae led to lower ETR, signaling compromised photochemical efficiency (Fukumoto et al., 2019). Additional impacts on photosynthetic performance have been reported in *Lessonia corrugata*, which exhibited reduced net productivity and increased erosion near its upper thermal threshold (James et al., 2023). Meanwhile, non-photochemical quenching (NPQ) often increases as a compensatory response, as in *Turbinaria ornata*, which raised NPQ to dissipate excess excitation energy under stress (Narrain et al., 2023).

Thermal stress also alters key biochemical traits in brown algae. For instance, *Sargassum horneri* exhibited significant reductions in chlorophylls above 30°C, indicating pigment degradation (Yong et al., 2021), while *Sargassum stenophyllum* showed declines in chlorophyll *a* after 5°C increase in temperature (Urrea-Victoria et al., 2020; Schmid et al., 2021) reported carotenoid adjustments and reductions in total pigment content in temperate kelps *Laminaria digitata* under heat stress, and *Macrocystis pyrifera* experienced losses in chlorophyll *c* and fucoxanthin under elevated temperatures (Mabin et al., 2019). Furthermore, recent work demonstrated that *L. spicata* exposed to thermal and light stress accumulates hydrogen peroxide (H_2_O_2_) and malondialdehyde (MDA), indicating oxidative damage and membrane destabilization (Celis-Plá et al., 2025). *L. spicata* (Suhr) (Laminariales, Phaeophyceae, Ochrophyta) is distributed along the temperate coast of Chile (29° to 41°S) and is one of the most ecologically important brown macroalgae in the Pacific Ocean (Buschmann et al., 2014; Gómez et al., 2016). It plays a foundational role in structuring intertidal and subtidal habitats, contributing to nutrient cycling, primary production, and habitat complexity.

In central Chile, where *L. spicata* is ecologically dominant, water temperatures can vary throughout the year, peaking during the summer months (December, January, and February) when temperatures range between 15–22 °C. However, significant increases in maximum temperatures have been recorded in recent years, attributable to global warming (Pujol et al., 2022; IPCC, 2023; Rojo-Garibaldi et al., 2023). These thermal anomalies such as the negative impacts observed on kelp beds in northern Chile (Tarapacá) during the recent El Niño event (Avilés and Parker, 2024), are altering distribution patterns. Notably, while *L. spicata* has historically dominated central Chile, recent findings document its expansion poleward (southward), reporting the first record of the species in the Sub-Antarctic Magellan Channels (Rosenfeld et al., 2019). This raises concerns about its long-term ecological viability and the potential loss of ecosystem services it supports. In this context, *in vivo* chlorophyll *a* fluorescence remains a valuable tool for detecting physiological impairment, as reductions in F_v_/F_m_ and ETR_max_ are widely used as indicators of photoinhibition and stress in kelps (Abdala-Díaz et al., 2006; Figueroa et al., 2016; Celis-Plá et al., 2016). These measures offer rapid, non-invasive insights into the photosynthetic status of macroalgae exposed to changing environments. Integrating these photophysiological metrics with biochemical traits such as pigment degradation, antioxidant capacity, and oxidative markers provides a comprehensive perspective on algal stress physiology.

Given regional warming trends and the increasing frequency of marine heat anomalies along central Chile, we tested a +3°C increment relative to 15°C to represent a realistic, short-term thermal stressor. This short-term stress is relevant because the degree of cellular damage and redox imbalance measured here is the mechanistic foundation that determines longer-term organismal fitness and demographic performance under chronic or repeated stress, as physiological responses underpin individual life-history traits and, consequently, population dynamics (Coleman, 2024). Our objective was strictly mechanistic: to quantify short-term photophysiological and biochemical responses of *L. spicata* to moderate warming under controlled conditions.

## Materials and methods

### Algae collection and experimental design

Thalli of *L. spicata* were collected in September 2023 from the rocky intertidal zone (upper intertidal) at Cochoa Beach, located north of Valparaíso Bay, Chile (32°57’19” S; 71°32’52” W). To avoid pseudoreplication, each replicate corresponded to a distinct thallus of *Lessonia spicata* collected from independent holdfasts at least 1 m within the sampling site. From each thallus, only one healthy blade segment was used per experimental unit, ensuring that all replicates represented independent biological individuals rather than subsamples from the same organism. A total of approximately 50 individuals were collected. The approximate sporophyte size ranged from 20 to 50 cm in length. During the sampling the sea surface temperature (SST) was 15 ± 1°C under low tide.

**Figure 1:**
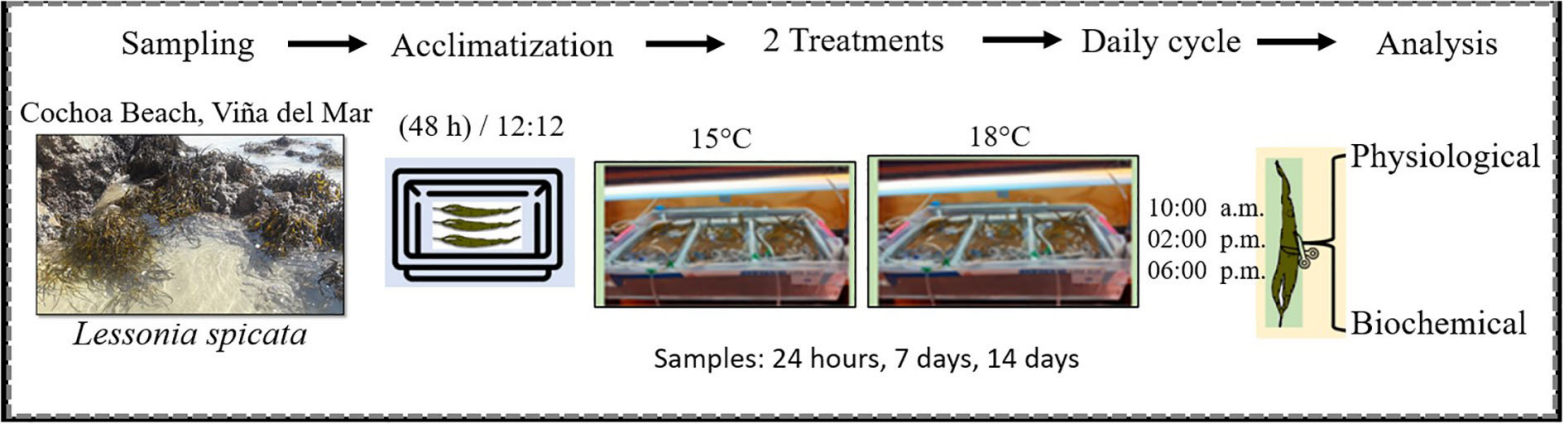
Explanatory diagram of the steps to follow during the experiment, from the sampling site to the collection of material for analysis.

The samples were transported in plastic containers under cooled (13-15°C) conditions to the Laboratory of Environmental Research (LACER) at the HUB Ambiental UPLA research center of Universidad de Playa Ancha, Chile. Upon arrival, thalli were maintained with constant aeration and periodic seawater exchange to stabilize temperature to (15°C) until the experiment began. Fronds from 10 to 11 different individuals were placed in each experimental unit (tank). During the entire experimental period, algae were exposed to a natural daily light regime simulating local conditions (PAR: 953 kJ m^-2^, UVA: 76.3 kJ m^-2^, UVB: 4.7 kJ m^-2^), under a 12:12 h light: dark photoperiod (**Figure 1**).

Two temperature treatments were applied: a control at (15°C) and a warming treatment of (18°C), as projected by the IPCC, 2023 scenario SSP2-4.5. The experimental unit for the temperature factor was the tank (n=3 per treatment), totaling 6 experimental units (n = 2 × 3). The temperature was regulated using a chiller system (Sunsun Chiller Mod. HYH 05D, Sensen, China). The tanks were assigned randomly to the two temperature treatments, and the temperature regulation was performed via an external water bath, ensuring no water circulation or exchange between the temperature treatments. Fronds were repositioned regularly to prevent self-shading, and fresh filtered seawater was changed every three days to avoid nutrient depletion and microbial overgrowth.

Sampling was performed at 10:00 h (local time) on day-1, day-7, and day-14. The selection of these time points was designed to capture both the immediate, acute physiological response (day-1) and the potential long-term response or compensatory acclimation (day-7; day-14). Photosynthetic parameters were measured *in situ*, on different adults’ fronds at each time point (days-1-7-14) to avoid time-series pseudo-replication and the influence of the measurement process. Algal tissue for biochemical and molecular analyses was collected from adult fronds using sterile scissors and immediately froze in liquid nitrogen to prevent degradation due to manipulation.

### Photosynthetic performance

*In vivo* chlorophyll *a* fluorescence was measured using a pulse amplitude modulated fluorometer (Junior-PAM, Walz GmbH, Germany).

The effective quantum yield (Δ*F/F*_m’_) Equation 1 was calculated from initial and maximum fluorescence under illuminated conditions, and maximum quantum yield (F_v_/F_m_) was determined after 15 minutes of dark adaptation (Figueroa et al., 2019; Celis-Plá et al., 2018, 2022, 2025).

The maximal electron transport rate (ETR_max_) (Equation 1) was estimated by exposing algal tissue to twelve different intensities of actinic light for 20 seconds during the rapid light curve (Schreiber et al., 2995).

(1) ETR (mmol electron m−2 s−1) = ΔF/Fm'* EPAR * A * FII

ETR parameters, including ETR_max_ and the initial slope of the ETR versus irradiance relationship (α_ETR_), were determined using the tangential model (Eilers and Peeters, 1988).

Non-photochemical quenching was calculated as follows (Celis-Plá et al., 2016) according to Equation 2:

(2) NPQ = (Fm − Fm') / Fm'  

The maximum non-photochemical quenching (NPQ_max_) was obtained by the tangential model contained in (Eilers and Peeters, 1988).

### Biochemical variables

All spectrophotometric measurements, including pigments, phenolics, antioxidant capacity, and malondialdehyde (MDA), were conducted using a microplate reader (SPECTROstar Nano, BMG Labtech, Offenburg, Germany), except for ROS fluorescence quantification, which was performed with a Cytation 5 instrument (Agilent BioTek, Santa Clara, USA).

Chlorophyll *a* and *c* were extracted from 20 mg of fresh algal tissue using 90% acetone, following (Ritchie, 2008). After 30 minutes of dark incubation at 4°C, samples were centrifuged at 16,200 g for 10 minutes. Absorbance was measured in microplates, and concentrations were calculated using the following Equations 3 and 4:

(3) Chl a = 11.47 × (A664 − A750)− 0.45 × (A630 − A750)

(4) Chl c = 22.679 × (A630 − A750)− 3.404 × (A664 − A750) 

Fucoxanthin concentration was determined from 25 mg of algal tissue using an extract in dimethyl sulfoxide (DMSO), following the modified protocol of (Seely et al., 1972) according to (Celis-Plá et al., 2918) (Equation 5). Absorbance was read at 480 nm and fucoxanthin concentration calculated using:

(5) Fx =[A480 − 0.722(A631 + A582 − 0.297 × A665) − 0.049 × A665]  / 130  

Total phenolic compounds were extracted from 250 mg of fresh-frozen algal tissue using 80% methanol and quantified using the Folin–Ciocalteu method with phloroglucinol as a standard, following (Celis-Plá et al., 2022). The antioxidant capacity was assessed via the DPPH (2,2-diphenyl-1-picrylhydrazyl) radical scavenging assay using 150 µL aliquots of the extract prepared from 250 mg of biomass, and results were expressed as μmol TEAC g^-1^ DW using Trolox as a standard (Álvarez-Gómez et al., 2017). Reactive oxygen species (ROS) production was measured from 30 mg of frozen algal tissue using a commercial detection kit (Sigma-Aldrich), following the manufacturer’s instructions. The tissue was extracted with HCL and reacted with the fluorophore probe. Fluorescence was read at 540/570 nm, and values were expressed as mmol g^-1^ DW. Lipid peroxidation was estimated from 50 mg of algal biomass via the quantification of malondialdehyde (MDA) using the thiobarbituric acid reactive substances (TBARS) method, following protocols described in Celis-Plá et al., 2025 and (Zúñiga et al., 2021), with absorbance measured at 532 nm. Results were expressed as ng MDA g^-1^ DW.

### Statistical analysis

We analyzed temperature (2 levels) × time (3 levels) with two-way ANOVA. For each response variable we report F, p, and partial η² with 95% CIs. Where interactions were significant, we provide simple effects with Hedges’ g and 95% CIs. Previously, the data were assessed for homogeneity and homoscedasticity of variance using Bartlett’s test, and student-Newman-Keuls *post hoc* tests were applied. All diagnostics and code are in **Supplementary Information**. The experimental unit and replication (n = … per treatment × time) are stated in each figure caption. Where significant interactions were observed, the. All statistical analyses were performed using R version 3.3.0 (R Cote Team).

Principal Coordinates Ordination (PCO) was conducted to integrate all photophysiological and biochemical variables and to visualize multivariate similarities among treatments. This complementary analysis allowed the identification of coordinated response patterns of *L. spicata* under control (15°C) and warming (+3°C) conditions. All variables—maximum quantum yield (F_v_/F_m_), maximum electron transport rate (ETR_max_), photosynthetic efficiency (α_ETR_), photosynthetic saturation irradiance (Ek_ETR_), non-photochemical quenching (NPQ_max_), chlorophyll *a* (Chl *a*), chlorophyll *c* (Chl *c*), fucoxanthin (FX), total phenolic compounds, antioxidant capacity (DPPH), reactive oxygen species (ROS), and malondialdehyde (MDA), Insoluble Phenolics compounds (PCI) and Soluble Phenolics Compounds (PCS) —were standardized by their maximum values to ensure comparability. The analysis was executed using the PRIMER v7 software package, following standard procedures for ecological datasets.

## Results

### Multivariate analysis (PCO)

**Figure 2:**
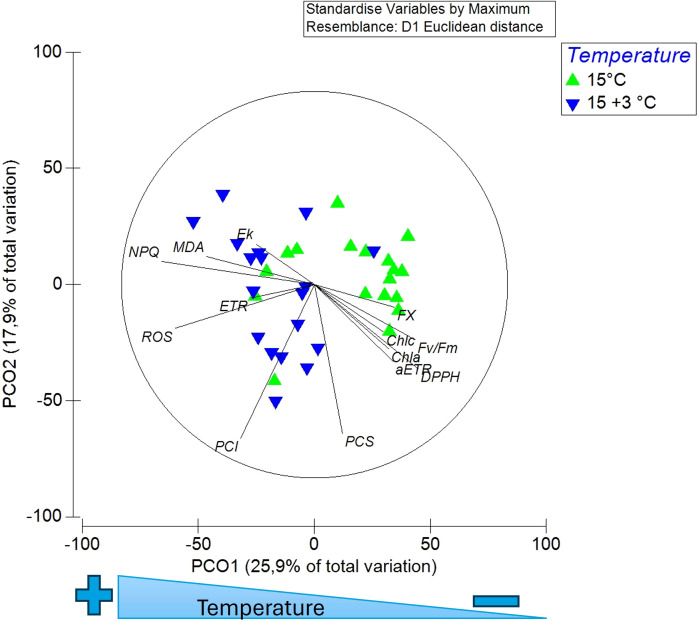
Principal Coordinates Ordination (PCO) of all photophysiological and biochemical variables. The ordination shows the separation between the 15°C (green triangles) and 18 °C (blue inverted triangles) treatments. Vectors indicate the contribution and direction of each variable. PCO1 and PCO2 explain 25.9% and 17.9% of the total variance, respectively.

The multivariate Principal Coordinates Ordination (PCO) analysis revealed a clear separation between treatments along the primary axis (PCO1), which explained 25.9% of the total variance (**Figure 2**). Samples exposed to 18 °C clustered towards the negative side of PCO1, associated with higher levels of non-photochemical quenching (NPQ), reactive oxygen species (ROS), and malondialdehyde (MDA). In contrast, control samples at 15 °C grouped on the positive side, characterized by higher values of maximum quantum yield (*F*_v_/*F*_m_), maximum electron transport rate (ETR_max_), photosynthetic efficiency (α_ETR_), and pigment contents (Chl *a*, Chl *c*, fucoxanthin), as well as greater antioxidant capacity (DPPH). The second axis (PCO2; 17.9% of total variance) captured minor within-treatment variability. Overall, this ordination underscores the consistent multivariate distinction between control and warming conditions, indicating that short-term exposure to +3 °C induces a coordinated decline in photochemical performance and redox homeostasis in *L. spicata*.

### Physiological responses

**Figure 3:**
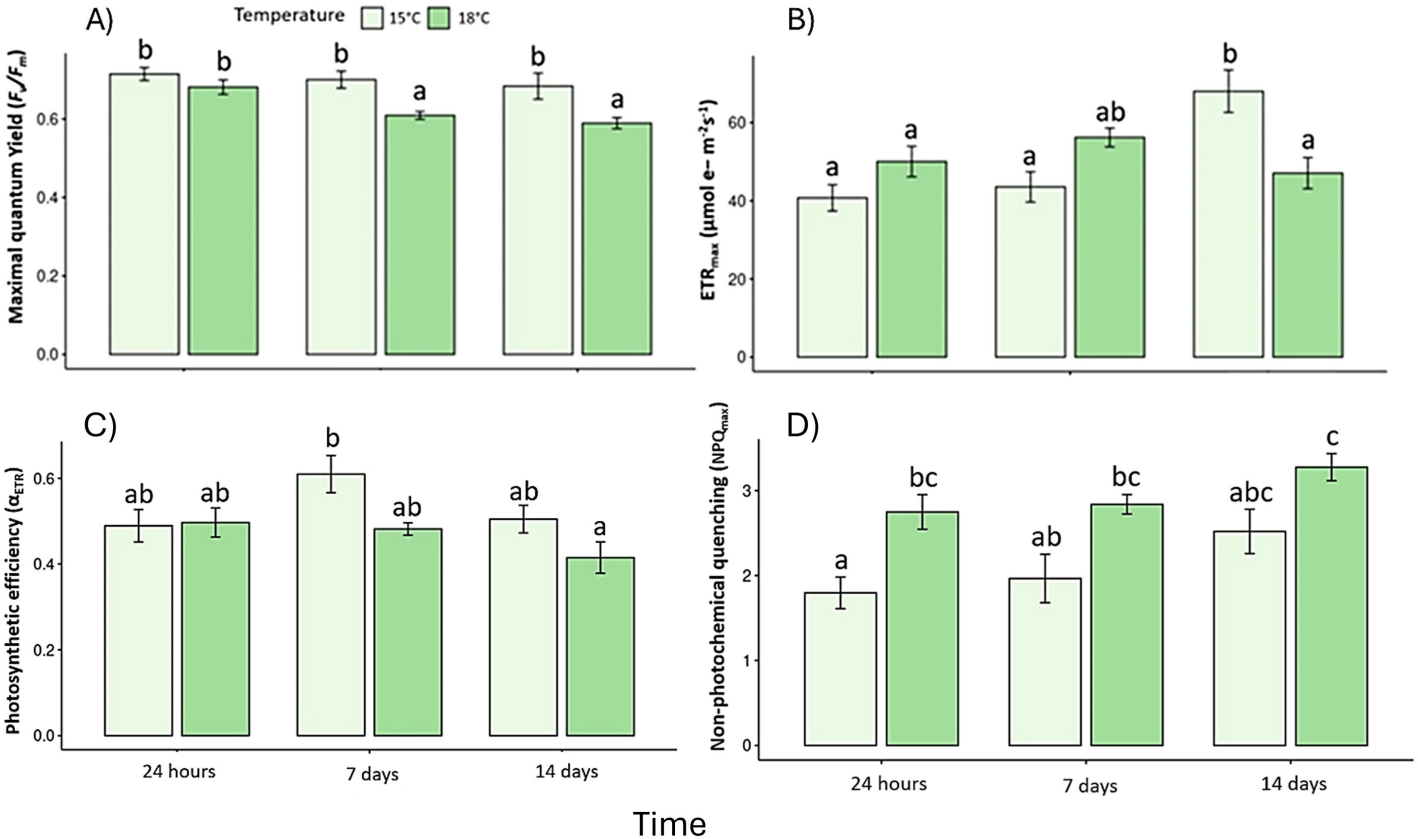
Photophysiological responses: **(A)** Maximal quantum yield (*F*_v_/*F*_m_), **(B)** maximal electron transport rate (ETR_max_). **(C)** Photosynthetic efficiency (α_ETR_) and **(D)** Maximal non-photochemical quenching (NPQ_max_). Lowercase letters denote significance after the SNK test.

Thermal stress produced consistent and statistically significant changes in the photophysiological performance of *L. spicata* (**Supplementary Table S1**). *F*_v_/*F*_m_ decreased progressively under 18°C treatment, with significant differences at days-7 and -14 relative to the control (F _(2, 30)_ = 18.9, *p* < 0.05, η² = 0.46). (**Figure 3A**). The corresponding Hedges’ *g* effect size for day-14 (–1.27, 95% CI = –1.98 to –0.56) confirms a strong impairment of PSII efficiency.

Similarly, ETR_max_ was markedly lower at 18°C by day-14 (F _(2, 30)_ = 0.07, p < 0.05, η² = 0.39), whereas control individuals exhibited a recovery trend (**Figure 3B**). The photosynthetic efficiency (α_ETR_) also declined at 18 °C, indicating reduced light-use capacity (F _(2, 30)_ = 5.66, p < 0.05) (**Figure 3C**). Although the photosynthetic saturation irradiance (Ek_ETR_) has no significant differences (data not shown). In contrast, maximum non-photochemical quenching (NPQ_max_) increased significantly at 18 °C from day-14 onward (F _(2, 30)_ = 25.8, p < 0.05, η² = 0.51) (**Figure 3D**), consistent with enhanced energy dissipation as a protective but transient mechanism. Correlation analysis revealed strong negative relationships between *F*_v_/*F*_m_ and NPQ_max_ (r = –0.78, p < 0.01) and between ETR_max_ and ROS (r = –0.71, p < 0.05), highlighting the functional coupling between photochemical inhibition and oxidative stress (**Figure 2**).

### Biochemical responses

**Figure 4:**
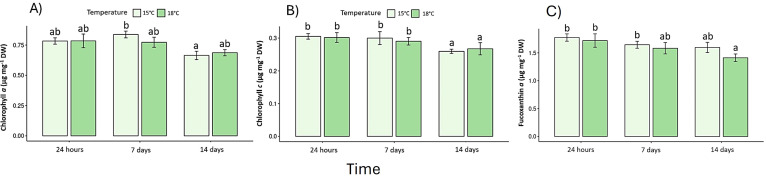
Photosynthetic pigments: **(A)** Chlorophyll *a* and **(B)** Chlorophyll *c* (c1+c2) and **(C)** Fucoxanthin pigment content. Lowercase letters denote significance after the SNK test.

**Figure 5:**
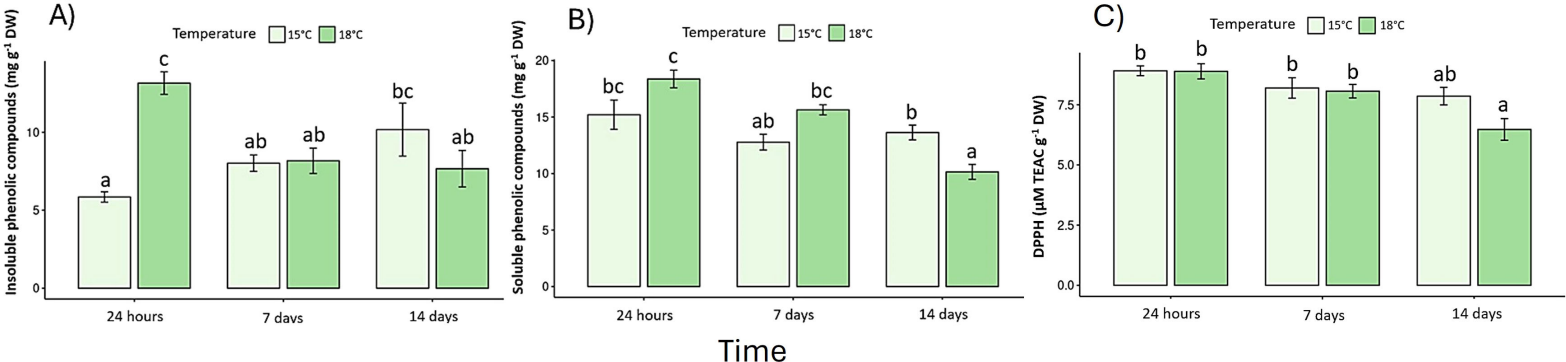
Phenolic compounds: **(A)** Insoluble phenolic content, **(B)** Soluble phenolic content, and **(C)** Total antioxidant capacity, measured by the DPPH assay and expressed as Trolox equivalents. Lowercase letters denote significance after the SNK test.

**Figure 6:**
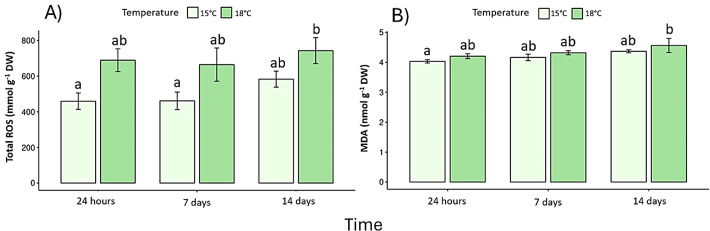
Oxidative stress markers: **(A)** Total Reactive Oxygen Species (ROS) and **(B)** lipid peroxidation (TBARS). Lowercase letters denote significance after the SNK test.

Pigment content declined under elevated temperature (**Supplementary Table S2**). Chlorophyll *a* decreased by 32% and chlorophyll *c* by 27% after 14 days at 18 °C (p < 0.05) (**Figures 4A, B**), whereas fucoxanthin declined by 23%, confirming pigment degradation (**Figure 4C**). In the same context, photoprotection as phenolic compounds exhibited a biphasic response: a transient rise at 24 h under 18 °C (early stress activation; F _(2, 30)_ = 13.7, p < 0.05) (**Figures 5A, B**), followed by a pronounced decrease on day-14. Antioxidant capacity (DPPH assay) dropped by 40% relative to the control (p < 0.05) (**Figure 5C**), while reactive oxygen species (ROS) (**Figure 6A**) and malondialdehyde (MDA) (**Figure 6B**) increased ~60% and 45%, respectively (p < 0.05) (**Figure 6**).

## Discussion

This study provides new experimental evidence of how *L. spicata* responds physiologically and biochemically to short-term thermal stress. Although integrative analyses of photophysiological and biochemical responses remain scarce for kelps from the southeastern Pacific, we simulated a realistic +3 °C increase relative to 15 °C – the mean summer temperature in central Chile – to quantify the immediate physiological and biochemical consequences of moderate warming under controlled laboratory conditions. The objective was explicitly mechanistic and time-bound, providing baseline data to interpret the cellular vulnerability of this species to near-future ocean warming. The multivariate analysis highlights the coordinated impairment of photosynthetic efficiency and redox balance in response to moderate warming. Here, the PCO analysis provides an integrated view of the multivariate stress response in *L. spicata* under moderate thermal stress. The clear separation of treatments along the first ordination axis demonstrates that even a +3 °C increment triggers a coherent shift in the species’ physiological state. The association of *F*_v_/*F*_m_, ETR_max_, α_ETR_, and pigment variables with the control treatment reflects efficient photochemical functioning and stable redox balance under ambient temperature. Conversely, the clustering of NPQ, ROS, and MDA vectors with the 18 °C samples highlight the convergence of photochemical down-regulation and oxidative stress as dominant processes under warming. This multivariate pattern supports the interpretation that short-term exposure to elevated temperature elicits a systemic response, coupling photophysiological impairment with biochemical redox disruption. Such integrative ordination evidence reinforces the notion that *L. spicata* exhibits limited short-term resilience to moderate thermal anomalies, aligning with experimental findings in other kelps subjected to comparable stress conditions (Li et al., 2020; Graiff and Karsten, 2021; Narrain et al., 2023).

Our results show a significant impairment of photosynthetic performance in *L. spicata* under moderate thermal stress. The reduction of maximum quantum yield (F_v_/F_m_) and maximal electron transport rate (ETR_max_) at 18°C on days-7 and -14, confirm sustained photoinhibition and damage to photosystem II. Such declines in photochemical efficiency have been widely observed in other brown macroalgae exposed to warming, including *Fucus serratus* (Figueroa et al., 2019), *Saccharina* spp. during summer anomalies (Li et al., 2020), and *Turbinaria ornata* under combined light-temperature stress (Narrain et al., 2023). The parallel reduction in α_ETR_ further indicates a decline in light-use efficiency, while the downward trend in Ek_ETR_ suggests constrained capacity to utilize high irradiance under heat exposure (Zúñiga et al., 2021). The marked increase in non-photochemical quenching (NPQ_max_) after 14 days reflects an enhanced dissipation of excess excitation energy, which acts as a short-term photoprotective mechanism. Nevertheless, sustained NPQ upregulation typically signals stress compensation rather than successful acclimation (Fukumoto et al., 2019), a pattern consistent with the seasonal NPQ behavior previously described for *L. spicata* across natural gradients (Zúñiga et al., 2021). Altogether, these physiological responses indicate that *L. spicata* operates near its upper thermal safety margin under moderate warm conditions. In its natural habitat, recurrent exposure to elevated temperatures and marine heatwaves could therefore push this species beyond its photophysiological compensation range, leading to declines in photosynthetic efficiency, carbon assimilation, and canopy recovery during summer peaks.

At the biochemical level, this study revealed consistent pigment degradation under warming. Chlorophyll *a* and *c* concentrations decreased significantly in 18°C treatment, particularly by day-14. These reductions indicate compromised photosynthetic structures, consistent with those observed in *S. horneri* at >30°C (Yong et al., 2021) and *S. stenophyllum* following a 5°C increase (Urrea-Victoria et al., 2020). The reduction of fucoxanthin - key for light harvesting and photoprotection - may further weaken the algae’s capacity to manage high light under warming (Mabin et al., 2019; Schmid et al., 2021). In the same context, phenolic compounds, which act as non-enzymatic antioxidants, displayed a biphasic response, a transient early increase at 24 h, indicative of rapid activation of defense pathways, followed by a steep decline over time This depletion coupled with the 40% reduction in overall antioxidant capacity (DPPH), suggests exhaustion of redox buffering mechanisms and the onset of oxidative stress (Cruces et al., 2013; Graiff and Karsten, 2021).

Comparable field patterns have been observed in *L. spicata*, where phenolic and antioxidant levels rise under moderate stress but collapse under extreme irradiance or warming (Celis-Plá et al., 2022). The increase in oxidative stress was evidenced by significantly elevated ROS and MDA levels in 18 °C treatment after 14 days, indicating lipid peroxidation and progressive membrane destabilization. These biochemical markers are reliable indicators of thermal damage in macroalgae, reflecting the breakdown of antioxidant defenses when stress thresholds are exceeded (Narrain et al., 2023; Celis-Plá et al., 2025). Similar oxidative impairments have been documented in *S. latissima* under thermal and nutrient stress (Li et al., 2020), and comparable redox imbalances have been reported in *S. horneri*, *S. stenophyllum*, and *L. digitata* under elevated temperatures, where disruptions in redox balance led to protein degradation, metabolic reallocation and compromised cellular integrity (Urrea-Victoria et al., 2020; Yong et al., 2021). In *S. mcclurei* and *F. vesiculosus*, even moderate warming has been shown to reduce antioxidant capacity and exacerbate ROS accumulation, particularly during prolonged exposures (Mabin et al., 2019; Graiff and Karsten, 2021). Together, these patterns indicate that the antioxidant system of *L. spicata* can transiently buffer stress but becomes depleted under sustained warming, reducing its capacity to mitigate oxidative injury. The convergence of pigment loss, oxidative stress, and increased NPQ reveals a shift from transient photoprotection toward persistent oxidative strain, a process likely to define the upper thermal limits of *L. spicata* under projected warming scenarios (Teagle and Smale, 2018; Schmid et al., 2021; Ji and Gao, 2021). Overall, the convergent photophysiological (lower F_v_/F_m_, ETR_max_, α_ETR_; higher NPQ_max_) and biochemical signals (lower chlorophylls/fucoxanthin, reduced phenolics/antioxidant capacity, higher ROS and MDA) indicate that *L. spicata* experienced short-term redox and photochemical stress under a +3 °C increment in controlled treatment. In natural populations, similar physiological disruption could reduce productivity, tissue renewal, and overall canopy persistence during recurrent heat anomalies. These findings align with projections of increased frequency and duration of marine heatwaves in the southeastern Pacific, often exceeding +1 °C for > 40 days (Carrasco et al., 2023; Pujol et al., 2023), which may expose *L. spicata* to cumulative physiological damage.

The sustained decline in photochemical performance (F_v_/F_m_, ETR_max_) and antioxidant capacity, alongside increased oxidative stress markers (ROS, MDA), suggest that the mechanisms normally used by this species to tolerate environmental variability are insufficient under prolonged moderate thermal stress. Future work should determine whether the short-term photophysiological and biochemical responses documented here persist over longer exposures and whether they co-vary with performance metrics measured independently (e.g., growth, survival, reproduction). Given that *L. spicata* spans ~29°S–48°S along the Chilean coast (González et al., 2016; Ramírez et al., 2023), future studies comparing northern and southern populations could reveal differential thermal tolerance and local adaptation. Such latitudinal contrasts may also help predict range retractions or shifts under progressive ocean warming. Considering ongoing reports of intensified marine heatwaves and regional warming trends (Carrasco et al., 2023; Pujol et al., 2023), both short- and long-term *in situ* experiments are warranted to evaluate acclimation and adaptive capacity under natural environmental fluctuations. Related work on seasonal variability in photosynthetic quotients under warming scenarios suggests useful methodological avenues for future assessments of primary production, bridging mechanistic physiology with ecosystem-scale consequences (Piticar, 2018; Demortier et al., 2022; Franke et al., 2024).

Overall, our endpoints reveal rapid and coordinated cellular responses to moderate warming, establishing a mechanistic foundation for future long-term studies that integrate physiological, demographic, and ecological dimensions of thermal tolerance in *L. spicata*, and by extension, the resilience of kelp forests along the Chilean coast.

## Conclusions

This study provides the mechanistic and integrated evidence that a +3 °C thermal increment—consistent with the IPCC SSP2-4.5 scenario—induces rapid declines in photochemical efficiency, pigment content, and antioxidant capacity in *L. spicata*, leading to oxidative stress within two weeks. By combining effect-size metrics and multivariate analyses, the present revision enhances the mechanistic interpretation of these short-term responses. Although the experiment was limited to a controlled day-14 exposure, the results establish a quantitative physiological baseline from which future long-term and omics-level investigations can be designed. These findings reinforce the ecological concern that projected ocean warming may threaten the persistence and ecosystem functions of this foundational kelp species along the southeastern Pacific coast.

## Data Availability

The original contributions presented in the study are included in the article/**Supplementary Material**. Further inquiries can be directed to the corresponding author.

## References

[B1] Abdala-Díaz R. T. Cabello-Pasini A. Pérez-Rodríguez E. Conde Álvarez R. M. Figueroa F. (2006). Daily and seasonal variations of optimum quantum yield and phenolic compounds in *Cystoseira tamariscifolia* (Phaeophyta). Mar. Biol. 148, 459–465. doi: 10.1007/s00227-005-0102-6

[B2] Álvarez-Gómez F. ,. F. Korbee N. Figueroa F. L. Abdala-Díaz R. T. (2017). Combined effects of UVR and nutrients on cell ultrastructure, photosynthesis and biochemistry in Gracilariopsis longissima (Gracilariales, Rhodophyta). Algal Res. 26, 190–202. doi: 10.1016/j.algal.2017.07.022

[B3] Avilés L. Parker U. (2024). Monitoreo de praderas de algas pardas y efectos del evento “El Niño” en la región de Tarapacá (Informe Técnico 3*)*. Centro Investigación Aplicada del Mar. S.A. Available online at: https://www.ciamchile.cl/wp-content/uploads/2024/03/Monitoreo-3-Praderas-Macroalgas_Febrero-2024_El-Nino-2023-24.CIAM_.pdf

[B4] Bell S. Y. Mabin C. J. T. Britton D. Johnson C. R. (2024). Season influences interspecific responses of canopy-forming kelps to warming and desiccation. Mar. Ecol. Prog. Ser. 740, 39–54. doi: 10.3354/meps13524

[B5] Buschmann A. H. Pereda S. V. Varela D. Rodríguez M. López A. González-Carvajal L. . (2014). Ecophysiological plasticity of annual populations of giant kelp (*Macrocystis pyrifera*) in a seasonally variable coastal environment in the Northern Patagonian Inner Seas of Southern Chile. J. Appl. Phycology 26, 837–847. doi: 10.1007/s10811-013-0070-z

[B6] Carneiro I. M. Paiva P. C. Bertocci I. Lorini M. L. de Széchy M. T. M. (2023). Distribution of a canopy-forming alga along the Western Atlantic Ocean under global warming: The importance of depth range. Mar. Environ. Res. 188, 106013. doi: 10.1016/j.marenvres.2023.106013, PMID: 37209442

[B7] Carrasco J. Montecinos A. Garreaud R. Ramos M. Pizarro C. Piticar A. . (2023). Marine heatwaves in the Southeastern Pacific: Intensity, duration, and future projections. Front. Mar. Sci. 10. doi: 10.3389/fmars.2023.1129276

[B8] Celis-Plá P. S. M. Brown M. T. Santillán-Sarmiento A. Korbee N. Sáez C. A. Figueroa F. L. (2018). Eco-physiological and metabolic responses to the interactive exposure to nutrients and excess copper in the brown macroalga Cystoseira tamariscifolia. Mar. pollut. Bull. 128, 214–222. doi: 10.1016/j.marpolbul.2018.01.005, PMID: 29571366

[B9] Celis-Plá P. S. M. Martínez B. Seabra R. Souza-Egipsy V. Quintano E. Lima F. P. (2016). Seasonal biochemical and photophysiological responses in the intertidal macroalga *Cystoseira tamariscifolia* (Ochrophyta). Mar. Environ. Res. 115, 89–97. doi: 10.1016/j.marenvres.2015.11.014, PMID: 26724873

[B10] Celis-Plá P. S. M. Navarrete C. E. Trabal A. Castro-Varela P. A. Figueroa F. L. Troncoso M. . (2025). Ecophysiological and biochemical responses of *L. spicata* to solar eclipse-induced light deprivation. Plants 14, 1810. doi: 10.3390/plants14121810, PMID: 40573797 PMC12196580

[B11] Celis-Plá P. S. M. Trabal A. Navarrete C. Troncoso M. Moenne F. Zúñiga A. . (2022). Daily changes on seasonal ecophysiological responses of the intertidal brown macroalga *L. spicata*: Implications of climate change. Front. Plant Sci. 13. doi: 10.3389/fpls.2022.941061, PMID: 36247624 PMC9554264

[B12] Coleman M. A. (2024). Algae in the Anthropocene: Managing, conserving, and utilizing algae in an era of rapid environmental change. J. Phycology 60, 1–3. doi: 10.1111/jpy.13409, PMID: 38010276

[B13] Coleman M. A. Wernberg T. (2017). Forgotten underwater forests: The key role of fucoid on Australian temperate reefs. Ecol. Evol. 7, 8406–8418. doi: 10.1002/ece3.3279r1, PMID: 29075458 PMC5648665

[B14] Cruces E. Huovinen P. Gómez I. (2013). Interactive effects of UV radiation and enhanced temperature on photosynthesis, phlorotannin induction and antioxidant activities of two sub-Antarctic brown algae. Mar. Biol. 160, 1–13. doi: 10.1007/s00227-012-2049-8

[B15] Demortier L. Garreaud R. Montecinos A. Pizarro O. Ramos M. Piticar A. (2022). The coastal ocean response to climate variability and change in the Eastern South Pacific: A synthesis. Front. Mar. Sci. 9. doi: 10.3389/fmars.2022.800325

[B16] Duarte C. M. Gattuso J. Hancke K. Gundersen H. Filbee-Dexter K. Pedersen M. . (2022). Global estimates of the extent and production of macroalgal forests’. Global Ecol. Biogeography 31, 1422–1439. doi: 10.1111/geb.13515or

[B17] Dubash N. K. (2020). Climate laws help reduce emissions. Nat. Climate Change 10, 709–710. doi: 10.1038/s41558-020-0853-6

[B18] Eilers P. H. C. Peeters J. C. H. (1988). A model for the relationship between light intensity and the rate of photosynthesis in phytoplankton. Ecol. Model. 42, 199–215. doi: 10.1016/0304-3800(88)90057-9

[B19] Figueroa F. L. Celis-Plá P. S. M. Martínez B. Korbee N. Trilla A. Arenas F. (2019). Yield losses and electron transport rate as indicators of thermal stress in *Fucus serratus* (Ochrophyta). Algal Res. 41, 101560. doi: 10.1016/j.algal.2019.101560

[B20] Figueroa F. L. Hermoso-Bentrán M. Celis-Pl. P. S. M. Bonomi-Barufi J. Álvarez-Gómez F. Korbee N. . (2016). Photosynthetic activity estimated as *in vivo* chlorophyll a fluorescence in calcareous red macroalgae. Cienc. Marinas 42, 139–155. doi: 10.7773/cm.v42i2.2587

[B21] Franke A. Bartsch I. Wiencke C. Graiff A. (2024). Varying photosynthetic quotients strongly influence net kelp primary production and seasonal differences increase under warming. Mar. Environ. Res. 195, 106157. doi: 10.1016/j.marenvres.2023.106157, PMID: 37690866

[B22] Fukumoto R. Yotsukura N. Endo H. Mikami K. (2019). Effect of photosynthetically active radiation and temperature on photosynthesis and growth in two brown algae, *Cladosiphon okamuranus* and Nemacystus decipiens. J. Appl. Phycology 31, 3687–3698. doi: 10.1007/s10811-018-1675-z

[B23] Gómez I. Español S. Velis K. Houvien P. (2016). Spatial distribution of phlorotannins and its relationship with photosynthetic UV tolerance and allocation of storage carbohydrates in blades of the kelp *Lessonia spicata*. Mar. Biol. 163, 110. doi: 10.1007/s00227-016-2891-1

[B24] González A. Rojas-Herrera R. Sáez C. A. Riosmena-Rodríguez R. Buschmann A. H. (2016). Frequency of chimerism in populations of the brown alga *Lessonia spicata* (Phaeophyceae, Laminariales) in Chile. J. Appl. Phycol. 28, 103–113. doi: 10.1007/s10811-015-0554-x

[B25] Graiff A. Karsten U. (2021). Antioxidative Properties of Baltic Sea Keystone Macroalgae (*Fucus vesiculosus*, Phaeophyceae) under Ocean Warming and Acidification in a Seasonally Varying Environment. Biology 10, 1330. doi: 10.3390/biology10121330, PMID: 34943245 PMC8698884

[B26] Gundersen H. Rinde E. Bekkby T. Hancke K. Gitmark JK and Christie H. (2021). Structure and standing stocks of kelp along multiple environmenta gradients and implications for ecosystem services. Front. Mar. Sci. 8. doi: 10.3389/fmars.2021.578629

[B27] Harley C. Randall Hughes A. Hultgren K. Miner B. Sorte C. Thornber C. . (2006). The impacts of climate change in coastal marine systems. Ecol. Lett. 9, 228–241. doi: 10.1111/j.1461-0248.2005.00871.x, PMID: 16958887

[B28] IPCC (2023). Summary for Policymakers. In: Climate Change 2023: Synthesis Report. Contribution of Working Groups I, II and III to the Sixth Assessment Report of the Intergovernmental Panel on Climate Change Core Writing Team Lee H. Romero J. (eds.) (Geneva, Switzerland: IPCC), pp. 1–34. doi: 10.59327/IPCC/AR6-9789291691647.001

[B29] James D. A. Johnson C. R. Mabin C. J. T. (2023). The endemic kelp *Lessonia corrugata* is being pushed to its thermal limits in a warming ocean. Proc. R. Soc. B: Biol. Sci. 290, 20232253. doi: 10.1098/rspb.2023.2253, PMID: 38228502 PMC10791590

[B30] Ji Y. Gao K. (2021). Effects of climate change factors on marine macroalgae: A review Advances in Marine Biology 88, 91–136. doi: 10.1016/bs.amb.2020.11.001, PMID: 34119047

[B31] Li H. Monteiro C. Heinrich S. Bartsch I. Valentin K. Harms L. . (2020). Responses of the kelp *Saccharina latissima* (Phaeophyceae) to the warming Arctic: from physiology to transcriptomics. Physiol. Plant 168, 5–26. doi: 10.1111/ppl.13009, PMID: 31267544

[B32] Mabin C. Johnson C. Wright J. (2019). Physiological response to temperature, light, and nitrates in the giant kelp Macrocystis pyrifera, from Tasmania, Australia. Mar. Ecol. Prog. Ser. 614, 1–19. doi: 10.3354/meps12900

[B33] Marambio J. Bischof K. (2021). Differential acclimation responses to irradiance and temperature in two co-occurring seaweed species in Arctic fjords. Polar Res. 40, 5702. doi: 10.33265/polar.v40.5702

[B34] Narrain K. Vairappan C. S. Sumiati S. (2023). Photosynthetic, phytochemical and antioxidant responses of three tropical brown algae to thermal and irradiance stress. Front. Mar. Sci. 10. doi: 10.3389/fmars.2021.578629

[B35] Piticar A. (2018). Changes in heatwaves in Chile. Glob. Planet. Change 169, 234–246. doi: 10.1016/j.gloplacha.2018.08.007

[B36] Pujol C. Montecinos A. Piticar A. Ramos M. Garreaud R. Pizarro C. (2023). Main drivers of marine heatwaves in the eastern South Pacific: Insights from 35 years of satellite data. Front. Mar. Sci. 10, 1129276. doi: 10.3389/fmars.2022.800325

[B37] Pujol C. Pérez-Santos I. Barth A. Alvera-Azcárate (2022). Marine heatwaves offshore central and South Chile: understanding forcing mechanisms during the years 2016-2017. Front. Mar. Sci. 9, 2022. doi: 10.3389/fmars.2022.800325

[B38] Ramírez C. Ávila A. Zamorano J. Troncoso M. (2023). Identificando oportunidades en el manejo del huiro negro (*Lessonia spicata*) para la adaptación al cambio climático (Santiago, Chile: Fundación Terram). Available online at: https://terram.cl/wp-content/uploads/2023/11/Informe-Huiro-Negro-2023.pdf (Accessed September 4, 2023).

[B39] R Core Team (2023). R: A language and environment for statistical computing (Vienna, Austria: R Foundation for Statistical Computing). Available online at: https://www.R-project.org/ (Accessed November 28, 2023).

[B40] Ritchie R. J. (2008). Universal chlorophyll equations for estimating chlorophylls a, b, c1 and *c2* and total chlorophylls in natural assemblages of photosynthetic organisms using acetone, methanol, or ethanol solvents. Photosynthetica 46, 115–126. doi: 10.1007/s11099-008-0019-7

[B41] Rojo-Garibaldi B. Contreras-López M. Giannerini S. Salas-de-León D. A. Vázquez-Guerra V. H. E. Cartwright J. (2023). Nonlinear time series analysis of coastal temperatures and El Niño–Southern Oscillation events in the eastern South Pacific, Earth Syst. Dynam. 14, 1125–1164. doi: 10.5194/esd-14-1125-2023

[B42] Rosenfeld S. Lagos N. A. Cerda M. (2019). *Lessonia spicata* as a foundation species: Implications for conservation and restoration. Rev. Biología Marina y Oceanografía. 54, 241–254. doi: 10.22370/rbmo.2019.54.2.1601

[B43] Schmid M. Gonçalves A. G. Seabra R. Faria J. Lima F. P. Nunes C. (2021). Acclimation potential and biochemical responses of four temperate macroalgae to light and future seasonal temperature scenarios. Mar. Environ. Res. 168, 105310. doi: 10.1016/j.marenvres.2021.105310, PMID: 33774470

[B44] Schreiber U. Bilger W. Neubauer C. (1995). Chlorophyll Fluorescence as a Nonintrusive Indicator for Rapid Assessment of *In Vivo* Photosynthesis in Ecophysiology of Photosynthesis (Berlin, Heidelberg: Springer Berlin Heidelberg), 49–70. doi: 10.1007/978-3-642-79354-7_3

[B45] Seely G. R. Duccan M. J. Vidiver W. E. (1972). Preparative and analytical extraction of pigments from brown algae with dimethyl sulfoxide. Mar. Biol. doi: 10.1007/BF00350754

[B46] Smale D. A. (2020). Impacts of ocean warming on kelp forest ecosystems. New Phytol. 225, 1447–1454. doi: 10.1111/nph.16107, PMID: 31400287

[B47] Teagle H. Smale D. A. (2018). Climate-driven substitution of habitat-forming species leads to reduced biodiversity within a temperate marine community. Diversity Distributions 24, 1367–1380. doi: 10.1111/ddi.12775

[B48] Trégarot E. D’Olivo J. Botelho A. Cabrito A. Cardoso G. Casal G. . (2024). Effects of climate change on marine coastal ecosystems -A review to guide research and management. Biol. Conserv. 289, 110394. doi: 10.1016/j.biocon.2023.110394

[B49] Urrea-Victoria V. Nardelli A. Floh E. Chow F. (2020). *Sargassum stenophyllum* (Fucale, Ochrophyta) responses to temperature short-term exposure: photosynthesis and chemical composition. Braz. J. Bot. 43, 733–745. doi: 10.1007/s40415-020-00639-y

[B50] Wernberg T. Krumhansl K. Filbee-Dexter K. Pedersen M. (2019). “ Status and trends for the world’s kelp forests’,” in World Seas: An Environmental Evaluation ( Elsevier), 57–78. doi: 10.1016/B978-0-12-805052-1.00003-6

[B51] Yong Y. S. Yusoff F. M. Shariff M. Fadhlullah Z. M. (2021). Effects of temperature and light on growth rate and photosynthetic characteristics of *Sargassum horneri*. J. Appl. Phycology 33, 1817–1828. doi: 10.1007/s10811-020-02174-5

[B52] Zúñiga A. Sáez C. A. Trabal A. Figueroa F. L. Pardo D. Navarrete C. . (2021). Seasonal photoacclimation and vulnerability patterns in the brown macroalga *Lessonia spicata* (Ochrophyta). Water 13, 6. doi: 10.3390/w13010006

