# Supplementary material for: Thermal stress impairs photophysiology and redox balance in the kelp Lessonia spicata
Source: Front Plant Sci. 2025 Dec 15;16:1727961. doi: 10.3389/fpls.2025.1727961 (PMC12745468; doi:10.3389/fpls.2025.1727961)
Supplement: Supplementary file 1 [file Table1.docx]

**Supplementary Material**

## **Table S1**. Two-way ANOVA results for physiological analysis of *Lessonia spicata* during an experiment with control temperatures (15°C) and a treatment (+3°C) for 14 days p<0.05**

|  | | ***Lessonia spicata*** | | | |
| --- | --- | --- | --- | --- | --- |
|  |  | ***df*** | ***MS*** | ***F*** | ***P*** |
| ***F*v/*F*m** | *Temperature (t)* | 1 | **0.020477** | **18.864** | ****** |
|  | *Exposure(e)* | 2 | **0.005539** | **5.103** | ****** |
|  | *t*e* | 2 | **0.001623** | **1.495** |  |
|  | *Res* | 30 | 0.001085 |  |  |
| **ETRmax** | *Temperature (t)* | 1 | **0.00241** | **0.301** |  |
|  | *Exposure(e)* | 2 | **0.02932** | **3.661** | ****** |
|  | *t*e* | 2 | **0.07071** | **8.830** | ****** |
|  | *Res* | 30 | 0.00801 |  |  |
| **αETR** | *Temperature (t)* | 1 | **0.03206** | **5.655** | ****** |
|  | *Exposure(e)* | 2 | **0.01819** | **3.209** |  |
|  | *t*e* | 2 | **0.01040** | **1.835** |  |
|  | *Res* | 30 | 0.00567 |  |  |
| **EkETR** | *Temperature (t)* | 1 | **0.00120** | **0.045** |  |
|  | *Exposure(e)* | 2 | **0.03167** | **1.17** |  |
|  | *t*e* | 2 | **0.04758** | **1.772** |  |
|  | *Res* | 30 | 0.02685 |  |  |
| **NPQmax** | *Temperature (t)* | 1 | **0.24140** | **25.764** | ****** |
|  | *Exposure(e)* | 2 | **0.04244** | **4.529** | ****** |
|  | *t*e* | 2 | **0.00374** | **0.399** |  |
|  | *Res* | 30 | 0.00937 |  |  |

*Res: Residual*

## **Table S2**. Two-way ANOVA results for biochemical analysis of *Lessonia spicata* during an experiment with control temperatures (15°C) and a treatment (+3°C) for 14 days p<0.05**

|  | | ***Lessonia spicata*** | | | |
| --- | --- | --- | --- | --- | --- |
|  |  | ***df*** | ***MS*** | ***F*** | ***P*** |
| **Chlorophyl *a*** | *Temperature (t)* | 1 | **0.000616** | **0.215** | ****** |
|  | *Exposure(e)* | 2 | **0.019939** | **6.965** |  |
|  | *t*e* | 2 | **0.002145** | **0.749** |  |
|  | *Res* | 30 | 0,002863 |  |  |
| **Chlorophyl *c*** | *Temperature (t)* | 1 | **0.000097** | **0.036** | ****** |
|  | *Exposure(e)* | 2 | **0.012949** | **4.843** |  |
|  | *t*e* | 2 | **0.000329** | **0.123** |  |
|  | *Res* | 30 | 0.002674 |  |  |
| **Fucoxanthin** | *Temperature (t)* | 1 | **0.015645** | **2.206** | ****** |
|  | *Exposure(e)* | 2 | **0.027397** | **3.864** |  |
|  | *t*e* | 2 | **0.002472** | **0.349** |  |
|  | *Res* | 30 | 0.007091 |  |  |
| **Phenolic compounds (Insoluble)** | *Temperature (t)* | 1 | **0.05734** | **4.826** | ****** |
|  | *Exposure(e)* | 2 | **0.00460** | **0.387** |  |
|  | *t*e* | 2 | **0.17737** | **14.927** |  |
|  | *Res* | 30 | 0.01188 |  |  |
| **Phenolic**  **compounds (Soluble)** | *Temperature (t)* | 1 | **0.00220** | **0.628** |  |
|  | *Exposure(e)* | 2 | **0.06919** | **19.770** | ****** |
|  | *t*e* | 2 | **0.04808** | **13.74** | ****** |
|  | *Res* | 30 | **0.00350** |  |  |
| **DPPH** | *Temperature (t)* | 1 | **0.008804** | **3.838** |  |

|  | *Exposure(e)* | 2 | **0.029512** | **12.864** | ****** |
| --- | --- | --- | --- | --- | --- |
|  | *t*e* | 2 | **0.006894** | **3.005** |  |
|  | *Res* | 30 | 0.002294 |  |  |
| **Total ROS** | *Temperature (t)* | 1 | **141.01** | **12.557** | ****** |
|  | *Exposure(e)* | 2 | **17.22** | **1.534** |  |
|  | *t*e* | 2 | **2.44** | **0.217** |  |
|  | *Res* | 30 | 11.23 |  |  |
| **MDA** | *Temperature (t)* | 1 | **0.002580** | **3.536** | ****** |
|  | *Exposure(e)* | 2 | **0.003552** | **4.868** |  |
|  | *t*e* | 2 | **0.000004** | **0.005** |  |
|  | *Res* | 30 | 0.000730 |  |  |

*Res: Residual*

## **Table S3.** Pearson correlation results for physiological analysis of *Lessonia spicata* during an experiment with control temperatures (15°C) and a treatment (+3°C) for 14 days p<0.05**

|  | ***Chla*** | ***Chlc*** | ***FX*** | ***ROS*** | ***MDA*** | ***FI*** | ***FS*** | ***DPPH*** | ***aETR*** | ***ETR*** | ***Ek*** | ***FvFm*** |
| --- | --- | --- | --- | --- | --- | --- | --- | --- | --- | --- | --- | --- |
| ***Chlc*** | *0.604** |  |  |  |  |  |  |  |  |  |  |  |
| ***FX*** | 0.362 | *0.509** |  |  |  |  |  |  |  |  |  |  |
| ***ROS*** | -0.116 | -0.079 | -0.21 |  |  |  |  |  |  |  |  |  |
| ***MDA*** | -0.139 | -0.34 | -0.347 | *0.389** |  |  |  |  |  |  |  |  |
| ***FI*** | 0.071 | -0.051 | -0.112 | 0.223 | 0.124 |  |  |  |  |  |  |  |
| ***FS*** | 0.157 | 0.275 | 0.23 | 0.055 | -0.246 | *0.429** |  |  |  |  |  |  |
| ***DPPH*** | 0.336 | 0.291 | 0.221 | -0.365 | *-0.413** | 0.119 | *0.459** |  |  |  |  |  |
| ***aETR*** | 0.255 | -0.026 | -0.011 | -0.087 | -0.236 | 0.112 | 0.223 | 0.198 |  |  |  |  |
| ***ETR*** | -0.297 | -0.184 | -0.154 | 0.162 | 0.24 | 0.165 | -0.002 | -0.021 | -0.195 |  |  |  |
| ***Ek*** | -0.268 | -0.151 | 0.071 | -0.029 | 0.288 | 0.095 | -0.031 | -0.071 | -0.343 | 0.144 |  |  |
| ***FvFm*** | 0.152 | 0.185 | *0.441** | -0.259 | -0.252 | 0.013 | 0.211 | *0.481** | 0.276 | 0.087 | -0.168 |  |
| ***NPQ*** | -0.175 | -0.196 | -0.253 | *0.374** | 0.322 | 0.224 | -0.183 | *-0.385** | *-0.403** | 0.082 | 0.124 | *-0.493** |

OBJ2

## 2024-12-05

**library**(stats) **library**(graphics) **library**(psych) **library**(readxl) **library**(agridat) **library**(ggplot2)

##

## Attaching package: 'ggplot2'

## The following objects are masked from 'package:psych': ##

## %+%, alpha

**library**(tidyr) **library**(dplyr)

##

## Attaching package: 'dplyr'

## The following objects are masked from 'package:stats':

##

## filter, lag

## The following objects are masked from 'package:base':

##

## intersect, setdiff, setequal, union

**library**(readxl)

AnalisisO2 <- **read_excel**("AnalisisO2.xlsx") AnalisisO2**$**Temperatura<-**as.factor**(AnalisisO2**$**Temperatura) AnalisisO2**$**Tiempo<-**as.factor**(AnalisisO2**$**Tiempo) AnalisisO2**$**Chla<-**as.numeric**(AnalisisO2**$**Chla) AnalisisO2**$**Chlc<-**as.numeric**(AnalisisO2**$**Chlc) AnalisisO2**$**FX<-**as.numeric**(AnalisisO2**$**FX)

AnalisisO2**$**ROS<-**as.numeric**(AnalisisO2**$**ROS) AnalisisO2**$**MDA<-**as.numeric**(AnalisisO2**$**MDA) AnalisisO2**$**FI<-**as.numeric**(AnalisisO2**$**FI) AnalisisO2**$**FS<-**as.numeric**(AnalisisO2**$**FS) AnalisisO2**$**DPPH<-**as.numeric**(AnalisisO2**$**DPPH) AnalisisO2**$**aETR<-**as.numeric**(AnalisisO2**$**aETR) AnalisisO2**$**ETR<-**as.numeric**(AnalisisO2**$**ETR) AnalisisO2**$**Ek<-**as.numeric**(AnalisisO2**$**Ek) AnalisisO2**$**fvfm<-**as.numeric**(AnalisisO2**$**fvfm) AnalisisO2**$**NPQ<-**as.numeric**(AnalisisO2**$**NPQ)

log_Chla <- **log10** (AnalisisO2 **$** Chla) sqrt_Chla <- **sqrt** (AnalisisO2 **$** Chla) log_Chlc <- **log10** (AnalisisO2 **$** Chlc) sqrt_Chlc <- **sqrt** (AnalisisO2 **$** Chlc) log_FX <- **log10** (AnalisisO2 **$** FX) sqrt_FX <- **sqrt** (AnalisisO2 **$** FX) log_ROS <- **log10** (AnalisisO2 **$** ROS) sqrt_ROS <- **sqrt** (AnalisisO2 **$** ROS) log_MDA <- **log10** (AnalisisO2 **$** MDA) sqrt_MDA <- **sqrt** (AnalisisO2 **$** MDA) log_FI <- **log10** (AnalisisO2 **$** FI) sqrt_FI <- **sqrt** (AnalisisO2 **$** FI) log_FS <- **log10** (AnalisisO2 **$** FS) sqrt_FS <- **sqrt** (AnalisisO2 **$** FS) log_DPPH <- **log10** (AnalisisO2 **$** DPPH) sqrt_DPPH <- **sqrt** (AnalisisO2 **$** DPPH) log_aETR <- **log10** (AnalisisO2 **$** aETR) sqrt_aETR <- **sqrt** (AnalisisO2 **$** aETR) log_ETR <- **log10** (AnalisisO2 **$** ETR) sqrt_ETR <- **sqrt** (AnalisisO2 **$** ETR) log_Ek <- **log10** (AnalisisO2 **$** Ek) sqrt_Ek <- **sqrt** (AnalisisO2 **$** Ek) log_fvfm <- **log10** (AnalisisO2 **$** fvfm) sqrt_fvfm <- **sqrt** (AnalisisO2 **$** fvfm) log_NPQ <- **log10** (AnalisisO2 **$** NPQ) sqrt_NPQ <- **sqrt** (AnalisisO2 **$** NPQ)

AnalisisO2 <- **cbind**(AnalisisO2,log_Chla) AnalisisO2 <- **cbind**(AnalisisO2,sqrt_Chla) AnalisisO2 <- **cbind**(AnalisisO2,log_Chlc) AnalisisO2 <- **cbind**(AnalisisO2,sqrt_Chlc) AnalisisO2 <- **cbind**(AnalisisO2,log_FX) AnalisisO2 <- **cbind**(AnalisisO2,sqrt_FX) AnalisisO2 <- **cbind**(AnalisisO2,log_ROS) AnalisisO2 <- **cbind**(AnalisisO2,sqrt_ROS) AnalisisO2 <- **cbind**(AnalisisO2,log_MDA) AnalisisO2 <- **cbind**(AnalisisO2,sqrt_MDA) AnalisisO2 <- **cbind**(AnalisisO2,log_FI) AnalisisO2 <- **cbind**(AnalisisO2,sqrt_FI) AnalisisO2 <- **cbind**(AnalisisO2,log_FS) AnalisisO2 <- **cbind**(AnalisisO2,sqrt_FS) AnalisisO2 <- **cbind**(AnalisisO2,log_DPPH) AnalisisO2 <- **cbind**(AnalisisO2,sqrt_DPPH) AnalisisO2 <- **cbind**(AnalisisO2,log_aETR) AnalisisO2 <- **cbind**(AnalisisO2,sqrt_aETR) AnalisisO2 <- **cbind**(AnalisisO2,log_ETR) AnalisisO2 <- **cbind**(AnalisisO2,sqrt_ETR) AnalisisO2 <- **cbind**(AnalisisO2,log_Ek) AnalisisO2 <- **cbind**(AnalisisO2,sqrt_Ek) AnalisisO2 <- **cbind**(AnalisisO2,log_fvfm) AnalisisO2 <- **cbind**(AnalisisO2,sqrt_fvfm) AnalisisO2 <- **cbind**(AnalisisO2,log_NPQ) AnalisisO2 <- **cbind**(AnalisisO2,sqrt_NPQ)

**hist**(AnalisisO2**$**sqrt_Chla)

0

2

4

0

2

4

6

# Histogram of AnalisisO2$sqrt_Chla

Frequency

8

10

## 0.70 0.75 0.80 0.85 0.90 0.95 1.00

AnalisisO2$sqrt_Chla

**hist**(AnalisisO2**$**sqrt_Chlc)

# Histogram of AnalisisO2$sqrt_Chlc

Frequency

6

8

10

## 0.50 0.55 0.60

AnalisisO2$sqrt_Chlc

**hist**(AnalisisO2**$**sqrt_FX)

0

2

4

0

1

2

3

4

# Histogram of AnalisisO2$sqrt_FX

Frequency

5

6

7

## 1.10 1.15 1.20 1.25 1.30 1.35 1.40 1.45

AnalisisO2$sqrt_FX

**hist**(AnalisisO2**$**sqrt_ROS)

# Histogram of AnalisisO2$sqrt_ROS

Frequency

6

8

## 15 20 25 30

AnalisisO2$sqrt_ROS

**hist**(AnalisisO2**$**sqrt_MDA)

0

1

2

3

4

0

2

4

6

# Histogram of AnalisisO2$sqrt_MDA

Frequency

8

10

12

## 2.0 2.1 2.2 2.3 2.4

AnalisisO2$sqrt_MDA

**hist**(AnalisisO2**$**sqrt_FI)

# Histogram of AnalisisO2$sqrt_FI

Frequency

5

6

7

## 2.5 3.0 3.5 4.0

AnalisisO2$sqrt_FI

**hist**(AnalisisO2**$**sqrt_FS)

0

2

4

0

2

4

# Histogram of AnalisisO2$sqrt_FS

Frequency

6

8

## 3.0 3.5 4.0 4.5

AnalisisO2$sqrt_FS

**hist**(AnalisisO2**$**sqrt_DPPH)

# Histogram of AnalisisO2$sqrt_DPPH

Frequency

6

8

## 2.4 2.6 2.8 3.0 3.2

AnalisisO2$sqrt_DPPH

**hist**(AnalisisO2**$**sqrt_aETR)

0

2

4

0

2

4

6

# Histogram of AnalisisO2$sqrt_aETR

Frequency

8

10

12

## 0.5 0.6 0.7 0.8 0.9

AnalisisO2$sqrt_aETR

**hist**(AnalisisO2**$**sqrt_ETR)

# Histogram of AnalisisO2$sqrt_ETR

Frequency

6

8

## 5 6 7 8 9

AnalisisO2$sqrt_ETR

**hist**(AnalisisO2**$**sqrt_Ek)

0

2

4

0

2

4

# Histogram of AnalisisO2$sqrt_Ek

Frequency

6

8

## 8 10 12 14 16

AnalisisO2$sqrt_Ek

**hist**(AnalisisO2**$**sqrt_fvfm)

# Histogram of AnalisisO2$sqrt_fvfm

Frequency

6

8

## 0.75 0.80 0.85

AnalisisO2$sqrt_fvfm

**hist**(AnalisisO2**$**sqrt_NPQ)

0

2

4

6

0

1

2

3

4

# Histogram of AnalisisO2$sqrt_NPQ

Frequency

5

6

7

## 1.0 1.2 1.4 1.6 1.8 2.0

AnalisisO2$sqrt_NPQ

**hist**(AnalisisO2**$**log_Chla)

# Histogram of AnalisisO2$log_Chla

Frequency

8

10

## −0.30 −0.25 −0.20 −0.15 −0.10 −0.05 0.00

AnalisisO2$log_Chla

**hist**(AnalisisO2**$**log_Chlc)

0

2

4

0

2

4

6

# Histogram of AnalisisO2$log_Chlc

Frequency

8

10

12

## −0.70 −0.65 −0.60 −0.55 −0.50 −0.45 −0.4

AnalisisO2$log_Chlc

**hist**(AnalisisO2**$**log_FX)

# Histogram of AnalisisO2$log_FX

Frequency

6

8

10

## 0.05 0.10 0.15 0.20 0.25 0.30 0.35

AnalisisO2$log_FX

**hist**(AnalisisO2**$**log_ROS)

0

5

0

2

4

6

# Histogram of AnalisisO2$log_ROS

Frequency

8

10

12

## 2.3 2.4 2.5 2.6 2.7 2.8 2.9 3.0

AnalisisO2$log_ROS

**hist**(AnalisisO2**$**log_MDA)

# Histogram of AnalisisO2$log_MDA

Frequency

10

15

## 0.60 0.65 0.70 0.75

AnalisisO2$log_MDA

**hist**(AnalisisO2**$**log_FI)

0

2

4

6

0

2

4

# Histogram of AnalisisO2$log_FI

Frequency

6

8

10

## 0.7 0.8 0.9 1.0 1.1 1.2 1.3

AnalisisO2$log_FI

**hist**(AnalisisO2**$**log_FS)

# Histogram of AnalisisO2$log_FS

Frequency

8

10

## 0.9 1.0 1.1 1.2 1.3

AnalisisO2$log_FS

**hist**(AnalisisO2**$**log_DPPH)

0

2

4

0

2

4

6

# Histogram of AnalisisO2$log_DPPH

Frequency

8

10

## 0.70 0.75 0.80 0.85 0.90 0.95 1.00

AnalisisO2$log_DPPH

**hist**(AnalisisO2**$**log_aETR)

# Histogram of AnalisisO2$log_aETR

Frequency

6

8

10

## −0.6 −0.5 −0.4 −0.3 −0.2 −0.

AnalisisO2$log_aETR

**hist**(AnalisisO2**$**log_ETR)

0

2

4

0

2

4

6

# Histogram of AnalisisO2$log_ETR

Frequency

8

10

12

## 1.4 1.5 1.6 1.7 1.8 1.9 2.0

AnalisisO2$log_ETR

**hist**(AnalisisO2**$**log_Ek)

# Histogram of AnalisisO2$log_Ek

Frequency

6

8

## 1.7 1.8 1.9 2.0 2.1 2.2 2.3 2.4

AnalisisO2$log_Ek

**hist**(AnalisisO2**$**log_fvfm)

0

2

4

6

0

1

2

3

4

# Histogram of AnalisisO2$log_fvfm

Frequency

5

6

7

## −0.25 −0.20 −0.15 −0.1

AnalisisO2$log_fvfm

**hist**(AnalisisO2**$**log_NPQ)

# Histogram of AnalisisO2$log_NPQ

Frequency

8

10

## 0.0 0.1 0.2 0.3 0.4 0.5 0.6

AnalisisO2$log_NPQ

*#Chla*

**bartlett.test**(Chla **~interaction**(Temperatura, Tiempo),data=AnalisisO2)

##

## Bartlett test of homogeneity of variances ##

## data: Chla by interaction(Temperatura, Tiempo)

## Bartlett's K-squared = 4.8965, df = 5, p-value = 0.4286

**bartlett.test**(**sqrt**(Chla)**~interaction**(Temperatura, Tiempo),data=AnalisisO2)

##

## Bartlett test of homogeneity of variances ##

## data: sqrt(Chla) by interaction(Temperatura, Tiempo) ## Bartlett's K-squared = 4.8955, df = 5, p-value = 0.4288

**bartlett.test**(**log**(Chla)**~interaction**(Temperatura, Tiempo),data=AnalisisO2)

##

## Bartlett test of homogeneity of variances ##

## data: log(Chla) by interaction(Temperatura, Tiempo)

## Bartlett's K-squared = 5.1425, df = 5, p-value = 0.3987

*#Chlc*

**bartlett.test**(Chlc **~interaction**(Temperatura, Tiempo),data=AnalisisO2)

##

## Bartlett test of homogeneity of variances ##

## data: Chlc by interaction(Temperatura, Tiempo)

## Bartlett's K-squared = 7.7909, df = 5, p-value = 0.1681

**bartlett.test**(**sqrt**(Chlc)**~interaction**(Temperatura, Tiempo),data=AnalisisO2)

##

## Bartlett test of homogeneity of variances ##

## data: sqrt(Chlc) by interaction(Temperatura, Tiempo) ## Bartlett's K-squared = 7.3803, df = 5, p-value = 0.1939

**bartlett.test**(**log**(Chlc)**~interaction**(Temperatura, Tiempo),data=AnalisisO2)

##

## Bartlett test of homogeneity of variances ##

## data: log(Chlc) by interaction(Temperatura, Tiempo)

## Bartlett's K-squared = 7.0997, df = 5, p-value = 0.2133

*#FX*

**bartlett.test**(FX **~interaction**(Temperatura, Tiempo),data=AnalisisO2)

##

## Bartlett test of homogeneity of variances ##

## data: FX by interaction(Temperatura, Tiempo)

## Bartlett's K-squared = 3.3752, df = 5, p-value = 0.6424

**bartlett.test**(**sqrt**(FX)**~interaction**(Temperatura, Tiempo),data=AnalisisO2)

##

## Bartlett test of homogeneity of variances ##

## data: sqrt(FX) by interaction(Temperatura, Tiempo)

## Bartlett's K-squared = 3.2341, df = 5, p-value = 0.664

**bartlett.test**(**log**(FX)**~interaction**(Temperatura, Tiempo),data=AnalisisO2)

##

## Bartlett test of homogeneity of variances ##

## data: log(FX) by interaction(Temperatura, Tiempo)

## Bartlett's K-squared = 3.1691, df = 5, p-value = 0.6739

*#ROS*

**bartlett.test**(ROS **~interaction**(Temperatura, Tiempo),data=AnalisisO2)

##

## Bartlett test of homogeneity of variances ##

## data: ROS by interaction(Temperatura, Tiempo)

## Bartlett's K-squared = 4.2104, df = 5, p-value = 0.5195

**bartlett.test**(**sqrt**(ROS)**~interaction**(Temperatura, Tiempo),data=AnalisisO2)

##

## Bartlett test of homogeneity of variances ##

## data: sqrt(ROS) by interaction(Temperatura, Tiempo)

## Bartlett's K-squared = 4.1277, df = 5, p-value = 0.5312

**bartlett.test**(**log**(ROS)**~interaction**(Temperatura, Tiempo),data=AnalisisO2)

##

## Bartlett test of homogeneity of variances ##

## data: log(ROS) by interaction(Temperatura, Tiempo)

## Bartlett's K-squared = 5.3269, df = 5, p-value = 0.3773

*#MDA*

**bartlett.test**(MDA **~interaction**(Temperatura, Tiempo),data=AnalisisO2)

##

## Bartlett test of homogeneity of variances ##

## data: MDA by interaction(Temperatura, Tiempo)

## Bartlett's K-squared = 18.173, df = 5, p-value = 0.002737

**bartlett.test**(**sqrt**(MDA)**~interaction**(Temperatura, Tiempo),data=AnalisisO2)

##

## Bartlett test of homogeneity of variances ##

## data: sqrt(MDA) by interaction(Temperatura, Tiempo)

## Bartlett's K-squared = 16.241, df = 5, p-value = 0.006189

**bartlett.test**(**log**(MDA)**~interaction**(Temperatura, Tiempo),data=AnalisisO2)

##

## Bartlett test of homogeneity of variances ##

## data: log(MDA) by interaction(Temperatura, Tiempo)

## Bartlett's K-squared = 14.505, df = 5, p-value = 0.0127

*#FI*

**bartlett.test**(FI **~interaction**(Temperatura, Tiempo),data=AnalisisO2)

##

## Bartlett test of homogeneity of variances ##

## data: FI by interaction(Temperatura, Tiempo)

## Bartlett's K-squared = 13.914, df = 5, p-value = 0.01617

**bartlett.test**(**sqrt**(FI)**~interaction**(Temperatura, Tiempo),data=AnalisisO2)

##

## Bartlett test of homogeneity of variances ##

## data: sqrt(FI) by interaction(Temperatura, Tiempo)

## Bartlett's K-squared = 11.529, df = 5, p-value = 0.04184

**bartlett.test**(**log**(FI)**~interaction**(Temperatura, Tiempo),data=AnalisisO2)

##

## Bartlett test of homogeneity of variances ##

## data: log(FI) by interaction(Temperatura, Tiempo)

## Bartlett's K-squared = 10.329, df = 5, p-value = 0.06644

*#FS*

**bartlett.test**(FS **~interaction**(Temperatura, Tiempo),data=AnalisisO2)

##

## Bartlett test of homogeneity of variances ##

## data: FS by interaction(Temperatura, Tiempo)

## Bartlett's K-squared = 6.0649, df = 5, p-value = 0.3

**bartlett.test**(**sqrt**(FS)**~interaction**(Temperatura, Tiempo),data=AnalisisO2)

##

## Bartlett test of homogeneity of variances ##

## data: sqrt(FS) by interaction(Temperatura, Tiempo)

## Bartlett's K-squared = 5.1378, df = 5, p-value = 0.3993

**bartlett.test**(**log**(FS)**~interaction**(Temperatura, Tiempo),data=AnalisisO2)

##

## Bartlett test of homogeneity of variances ##

## data: log(FS) by interaction(Temperatura, Tiempo)

## Bartlett's K-squared = 5.6101, df = 5, p-value = 0.346

*#DPPH*

**bartlett.test**(DPPH **~interaction**(Temperatura, Tiempo),data=AnalisisO2)

##

## Bartlett test of homogeneity of variances ##

## data: DPPH by interaction(Temperatura, Tiempo)

## Bartlett's K-squared = 3.5975, df = 5, p-value = 0.6087

**bartlett.test**(**sqrt**(DPPH)**~interaction**(Temperatura, Tiempo),data=AnalisisO2)

##

## Bartlett test of homogeneity of variances ##

## data: sqrt(DPPH) by interaction(Temperatura, Tiempo) ## Bartlett's K-squared = 4.5682, df = 5, p-value = 0.4708

**bartlett.test**(**log**(DPPH)**~interaction**(Temperatura, Tiempo),data=AnalisisO2)

##

## Bartlett test of homogeneity of variances ##

## data: log(DPPH) by interaction(Temperatura, Tiempo)

## Bartlett's K-squared = 5.7952, df = 5, p-value = 0.3267

*#aETR*

**bartlett.test**(aETR **~interaction**(Temperatura, Tiempo),data=AnalisisO2)

##

## Bartlett test of homogeneity of variances ##

## data: aETR by interaction(Temperatura, Tiempo)

## Bartlett's K-squared = 4.9513, df = 5, p-value = 0.4218

**bartlett.test**(**sqrt**(aETR)**~interaction**(Temperatura, Tiempo),data=AnalisisO2)

##

## Bartlett test of homogeneity of variances ##

## data: sqrt(aETR) by interaction(Temperatura, Tiempo) ## Bartlett's K-squared = 4.7573, df = 5, p-value = 0.4462

**bartlett.test**(**log**(aETR)**~interaction**(Temperatura, Tiempo),data=AnalisisO2)

##

## Bartlett test of homogeneity of variances ##

## data: log(aETR) by interaction(Temperatura, Tiempo)

## Bartlett's K-squared = 5.6088, df = 5, p-value = 0.3462

*#ETR*

**bartlett.test**(ETR **~interaction**(Temperatura, Tiempo),data=AnalisisO2)

##

## Bartlett test of homogeneity of variances ##

## data: ETR by interaction(Temperatura, Tiempo)

## Bartlett's K-squared = 3.0976, df = 5, p-value = 0.6849

**bartlett.test**(**sqrt**(ETR)**~interaction**(Temperatura, Tiempo),data=AnalisisO2)

##

## Bartlett test of homogeneity of variances ##

## data: sqrt(ETR) by interaction(Temperatura, Tiempo)

## Bartlett's K-squared = 2.5949, df = 5, p-value = 0.7621

**bartlett.test**(**log**(ETR)**~interaction**(Temperatura, Tiempo),data=AnalisisO2)

##

## Bartlett test of homogeneity of variances ##

## data: log(ETR) by interaction(Temperatura, Tiempo)

## Bartlett's K-squared = 3.3727, df = 5, p-value = 0.6427

*#Ek*

**bartlett.test**(Ek **~interaction**(Temperatura, Tiempo),data=AnalisisO2)

##

## Bartlett test of homogeneity of variances ##

## data: Ek by interaction(Temperatura, Tiempo)

## Bartlett's K-squared = 3.4857, df = 5, p-value = 0.6255

**bartlett.test**(**sqrt**(Ek)**~interaction**(Temperatura, Tiempo),data=AnalisisO2)

##

## Bartlett test of homogeneity of variances ##

## data: sqrt(Ek) by interaction(Temperatura, Tiempo)

## Bartlett's K-squared = 3.5746, df = 5, p-value = 0.6121

**bartlett.test**(**log**(Ek)**~interaction**(Temperatura, Tiempo),data=AnalisisO2)

##

## Bartlett test of homogeneity of variances ##

## data: log(Ek) by interaction(Temperatura, Tiempo)

## Bartlett's K-squared = 4.3883, df = 5, p-value = 0.495

*#fvfm*

**bartlett.test**(fvfm **~interaction**(Temperatura, Tiempo),data=AnalisisO2)

##

## Bartlett test of homogeneity of variances ##

## data: fvfm by interaction(Temperatura, Tiempo)

## Bartlett's K-squared = 7.6882, df = 5, p-value = 0.1743

**bartlett.test**(**sqrt**(fvfm)**~interaction**(Temperatura, Tiempo),data=AnalisisO2)

##

## Bartlett test of homogeneity of variances ##

## data: sqrt(fvfm) by interaction(Temperatura, Tiempo) ## Bartlett's K-squared = 7.2983, df = 5, p-value = 0.1994

**bartlett.test**(**log**(fvfm)**~interaction**(Temperatura, Tiempo),data=AnalisisO2)

##

## Bartlett test of homogeneity of variances ##

## data: log(fvfm) by interaction(Temperatura, Tiempo)

## Bartlett's K-squared = 7.0197, df = 5, p-value = 0.2192

*#NPQ*

**bartlett.test**(NPQ **~interaction**(Temperatura, Tiempo),data=AnalisisO2)

##

## Bartlett test of homogeneity of variances ##

## data: NPQ by interaction(Temperatura, Tiempo)

## Bartlett's K-squared = 4.6811, df = 5, p-value = 0.456

**bartlett.test**(**sqrt**(NPQ)**~interaction**(Temperatura, Tiempo),data=AnalisisO2)

##

## Bartlett test of homogeneity of variances ##

## data: sqrt(NPQ) by interaction(Temperatura, Tiempo)

## Bartlett's K-squared = 6.2961, df = 5, p-value = 0.2785

**bartlett.test**(**log**(NPQ)**~interaction**(Temperatura, Tiempo),data=AnalisisO2)

##

## Bartlett test of homogeneity of variances ##

## data: log(NPQ) by interaction(Temperatura, Tiempo)

## Bartlett's K-squared = 8.8283, df = 5, p-value = 0.1161

*#Chla*

AnovaChla<-**aov**(log_Chla**~**Temperatura**+**Tiempo**+**Temperatura*****Tiempo,data=AnalisisO2)

**summary**(AnovaChla)

| ## | Df | Sum Sq | Mean Sq F value | Pr(>F) |
| --- | --- | --- | --- | --- |
| ## Temperatura | 1 | 0.00062 | 0.000616 0.215 | 0.64610 |
| ## Tiempo | 2 | 0.03988 | 0.019939 6.965 | 0.00328 ** |
| ## Temperatura:Tiempo | 2 | 0.00429 | 0.002145 0.749 | 0.48129 |
| ## Residuals | 30 | 0.08589 | 0.002863 |  |
| ## --- |  |  |  |  |

## Signif. codes: 0 '***' 0.001 '**' 0.01 '*' 0.05 '.' 0.1 ' ' 1

AnovaChla2<-**aov**(sqrt_Chla**~**Temperatura**+**Tiempo**+**Temperatura*****Tiempo,data=AnalisisO2)

**summary**(AnovaChla2)

| ## | Df | Sum Sq | Mean Sq F value | Pr(>F) |
| --- | --- | --- | --- | --- |
| ## Temperatura | 1 | 0.00059 | 0.000592 0.213 | 0.64766 |
| ## Tiempo | 2 | 0.03899 | 0.019495 7.018 | 0.00316 ** |
| ## Temperatura:Tiempo | 2 | 0.00412 | 0.002061 0.742 | 0.48466 |
| ## Residuals | 30 | 0.08334 | 0.002778 |  |
| ## --- |  |  |  |  |

## Signif. codes: 0 '***' 0.001 '**' 0.01 '*' 0.05 '.' 0.1 ' ' 1

*#Chlc*

AnovaChlc<-**aov**(log_Chlc**~**Temperatura**+**Tiempo**+**Temperatura*****Tiempo,data=AnalisisO2)

**summary**(AnovaChlc)

| ## | Df | Sum Sq | Mean Sq F value Pr(>F) |
| --- | --- | --- | --- |
| ## Temperatura | 1 | 0.00010 | 0.000097 0.036 0.851 |
| ## Tiempo | 2 | 0.02590 | 0.012949 4.843 0.015 * |
| ## Temperatura:Tiempo | 2 | 0.00066 | 0.000329 0.123 0.885 |
| ## Residuals | 30 | 0.08021 | 0.002674 |
| ## --- |  |  |  |

## Signif. codes: 0 '***' 0.001 '**' 0.01 '*' 0.05 '.' 0.1 ' ' 1

AnovaChlc2<-**aov**(sqrt_Chlc**~**Temperatura**+**Tiempo**+**Temperatura*****Tiempo,data=AnalisisO2)

**summary**(AnovaChlc2)

| ## | Df | Sum Sq | Mean Sq | F value Pr(>F) |
| --- | --- | --- | --- | --- |
| ## Temperatura | 1 | 0.000031 | 0.000031 | 0.030 0.8636 |
| ## Tiempo | 2 | 0.009546 | 0.004773 | 4.628 0.0177 * |
| ## Temperatura:Tiempo | 2 | 0.000324 | 0.000162 | 0.157 0.8553 |
| ## Residuals | 30 | 0.030939 | 0.001031 |  |
| ## --- |  |  |  |  |

## Signif. codes: 0 '***' 0.001 '**' 0.01 '*' 0.05 '.' 0.1 ' ' 1

*#FX*

AnovaFX<-**aov**(log_FX**~**Temperatura**+**Tiempo**+**Temperatura*****Tiempo,data=AnalisisO2)

**summary**(AnovaFX)

| ## | Df | Sum Sq | Mean Sq F value Pr(>F) |
| --- | --- | --- | --- |
| ## Temperatura | 1 | 0.00808 | 0.008081 2.414 0.1308 |
| ## Tiempo | 2 | 0.02557 | 0.012783 3.818 0.0333 * |
| ## Temperatura:Tiempo | 2 | 0.00232 | 0.001159 0.346 0.7102 |
| ## Residuals | 30 | 0.10044 | 0.003348 |
| ## --- |  |  |  |

## Signif. codes: 0 '***' 0.001 '**' 0.01 '*' 0.05 '.' 0.1 ' ' 1

AnovaFX2<-**aov**(sqrt_FX**~**Temperatura**+**Tiempo**+**Temperatura*****Tiempo,data=AnalisisO2)

**summary**(AnovaFX2)

| ## | Df | Sum Sq | Mean Sq F value Pr(>F) |
| --- | --- | --- | --- |
| ## Temperatura | 1 | 0.01564 | 0.015645 2.206 0.1479 |
| ## Tiempo | 2 | 0.05479 | 0.027397 3.864 0.0321 * |
| ## Temperatura:Tiempo | 2 | 0.00494 | 0.002472 0.349 0.7085 |
| ## Residuals | 30 | 0.21272 | 0.007091 |
| ## --- |  |  |  |

## Signif. codes: 0 '***' 0.001 '**' 0.01 '*' 0.05 '.' 0.1 ' ' 1

*#ROS*

AnovaROS<-**aov**(log_ROS**~**Temperatura**+**Tiempo**+**Temperatura*****Tiempo,data=AnalisisO2)

**summary**(AnovaROS)

| ## | Df | Sum Sq | Mean Sq F value | Pr(>F) |
| --- | --- | --- | --- | --- |
| ## Temperatura | 1 | 0.1737 | 0.17372 10.475 | 0.00295 ** |
| ## Tiempo | 2 | 0.0526 | 0.02630 1.586 | 0.22140 |
| ## Temperatura:Tiempo | 2 | 0.0092 | 0.00461 0.278 | 0.75929 |
| ## Residuals | 30 | 0.4975 | 0.01658 |  |
| ## --- |  |  |  |  |

## Signif. codes: 0 '***' 0.001 '**' 0.01 '*' 0.05 '.' 0.1 ' ' 1

AnovaROS2<-**aov**(sqrt_ROS**~**Temperatura**+**Tiempo**+**Temperatura*****Tiempo,data=AnalisisO2)

**summary**(AnovaROS2)

| ## | Df Sum Sq Mean Sq F value | Pr(>F) |
| --- | --- | --- |
| ## Temperatura | 1 141.0 141.01 12.557 | 0.00132 ** |
| ## Tiempo | 2 34.4 17.22 1.534 | 0.23221 |
| ## Temperatura:Tiempo | 2 4.9 2.44 0.217 | 0.80610 |
| ## Residuals | 30 336.9 11.23 |  |
| ## --- |  |  |

## Signif. codes: 0 '***' 0.001 '**' 0.01 '*' 0.05 '.' 0.1 ' ' 1

*#MDA*

AnovaMDA<-**aov**(log_MDA**~**Temperatura**+**Tiempo**+**Temperatura*****Tiempo,data=AnalisisO2)

**summary**(AnovaMDA)

| ## | Df | Sum Sq | Mean Sq | F value Pr(>F) |
| --- | --- | --- | --- | --- |
| ## Temperatura | 1 | 0.002580 | 0.002580 | 3.536 0.0698 . |
| ## Tiempo | 2 | 0.007104 | 0.003552 | 4.868 0.0148 * |
| ## Temperatura:Tiempo | 2 | 0.000007 | 0.000004 | 0.005 0.9949 |
| ## Residuals | 30 | 0.021889 | 0.000730 |  |
| ## --- |  |  |  |  |
| ## Signif. codes: 0 '***' 0.001 '**' 0.01 | | | | '*' 0.05 '.' 0.1 ' ' 1 |
| AnovaMDA2<-**aov**(sqrt_MDA**~**Temperatura**+**Tiempo**+**Temperatura*****Tiempo,data=AnalisisO2)  **summary**(AnovaMDA2) | | | | |
| ## | Df | Sum Sq | Mean Sq F value Pr(>F) | |
| ## Temperatura | 1 | 0.01523 | 0.015230 3.399 0.0751 . | |
| ## Tiempo | 2 | 0.04171 | 0.020854 4.655 0.0173 * | |
| ## Temperatura:Tiempo | 2 | 0.00005 | 0.000024 0.005 0.9947 | |
| ## Residuals | 30 | 0.13440 | 0.004480 | |
| ## --- |  |  |  | |

## Signif. codes: 0 '***' 0.001 '**' 0.01 '*' 0.05 '.' 0.1 ' ' 1

*#FI*

AnovaFI<-**aov**(log_FI**~**Temperatura**+**Tiempo**+**Temperatura*****Tiempo,data=AnalisisO2)

**summary**(AnovaFI)

| ## | Df | Sum Sq | Mean Sq | F value | Pr(>F) |
| --- | --- | --- | --- | --- | --- |
| ## Temperatura | 1 | 0.0573 | 0.05734 | 4.826 | 0.0359 * |
| ## Tiempo | 2 | 0.0092 | 0.00460 | 0.387 | 0.6825 |
| ## Temperatura:Tiempo | 2 | 0.3547 | 0.17737 | 14.927 | 3.17e-05 *** |
| ## Residuals | 30 | 0.3565 | 0.01188 |  |  |
| ## --- |  |  |  |  |  |

## Signif. codes: 0 '***' 0.001 '**' 0.01 '*' 0.05 '.' 0.1 ' ' 1

AnovaFI2<-**aov**(sqrt_FI**~**Temperatura**+**Tiempo**+**Temperatura*****Tiempo,data=AnalisisO2)

**summary**(AnovaFI2)

| ## | Df | Sum Sq | Mean Sq | F value | Pr(>F) |
| --- | --- | --- | --- | --- | --- |
| ## Temperatura | 1 | 0.673 | 0.6734 | 4.599 | 0.0402 * |
| ## Tiempo | 2 | 0.205 | 0.1023 | 0.699 | 0.5052 |
| ## Temperatura:Tiempo | 2 | 4.179 | 2.0893 | 14.269 | 4.42e-05 *** |
| ## Residuals | 30 | 4.393 | 0.1464 |  |  |
| ## --- |  |  |  |  |  |
| ## Signif. codes: 0 '***' 0.001 | | | '**' 0.01 '*' 0.05 '.' 0.1 ' ' 1 | | |

*#FS*

AnovaFS<-**aov**(log_FS**~**Temperatura**+**Tiempo**+**Temperatura*****Tiempo,data=AnalisisO2)

**summary**(AnovaFS)

| ## | Df | Sum Sq | Mean Sq F value Pr(>F) | |
| --- | --- | --- | --- | --- |
| ## Temperatura | 1 | 0.00220 | 0.00220 0.628 0.434 | |
| ## Tiempo | 2 | 0.13837 | 0.06919 19.770 3.34e-06 *** | |
| ## Temperatura:Tiempo | 2 | 0.09616 | 0.04808 13.740 5.81e-05 *** | |
| ## Residuals | 30 | 0.10499 | 0.00350 | |
| ## --- |  |  |  | |
| ## Signif. codes: 0 '***' 0.001 | | | '**' 0.01 '*' 0.05 '.' 0.1 ' ' 1 | |
| AnovaFS2<-**aov**(sqrt_FS**~**Temperatura**+**Tiempo**+**Temperatura*****Tiempo,data=AnalisisO2)  **summary**(AnovaFS2) | | | | |
| ## | Df | Sum Sq | Mean Sq F value | Pr(>F) |
| ## Temperatura | 1 | 0.0744 | 0.0744 1.147 | 0.292752 |
| ## Tiempo | 2 | 2.5363 | 1.2681 19.559 | 3.65e-06 *** |
| ## Temperatura:Tiempo | 2 | 1.6246 | 0.8123 12.529 | 0.000111 *** |
| ## Residuals | 30 | 1.9451 | 0.0648 |  |
| ## --- |  |  |  |  |
| ## Signif. codes: 0 '***' 0.001 | | | '**' 0.01 '*' 0.05 '.' 0.1 ' ' 1 | |
| *#DPPH*  AnovaDPPH<-**aov**(log_DPPH**~**Temperatura**+**Tiempo**+**Temperatura*****Tiempo,data=AnalisisO2)  **summary**(AnovaDPPH) | | | | |
| ## | Df | Sum Sq | Mean Sq F value Pr(>F) | |
| ## Temperatura | 1 | 0.00880 | 0.008804 3.838 0.0595 . | |
| ## Tiempo | 2 | 0.05902 | 0.029512 12.864 9.24e-05 *** | |
| ## Temperatura:Tiempo | 2 | 0.01379 | 0.006894 3.005 0.0646 . | |
| ## Residuals | 30 | 0.06883 | 0.002294 | |
| ## --- |  |  |  | |
| ## Signif. codes: 0 '***' 0.001 | | | '**' 0.01 '*' 0.05 '.' 0.1 ' ' 1 | |
| AnovaDPPH2<-**aov**(sqrt_DPPH**~**Temperatura**+**Tiempo**+**Temperatura*****Tiempo,data=AnalisisO2)  **summary**(AnovaDPPH2) | | | | |
| ## | Df | Sum Sq | Mean Sq F value | Pr(>F) |
| ## Temperatura | 1 | 0.0828 | 0.08278 3.531 | 0.069995 . |
| ## Tiempo | 2 | 0.5921 | 0.29605 12.626 | 0.000105 *** |
| ## Temperatura:Tiempo | 2 | 0.1246 | 0.06230 2.657 | 0.086629 . |
| ## Residuals | 30 | 0.7034 | 0.02345 |  |
| ## --- |  |  |  |  |

## Signif. codes: 0 '***' 0.001 '**' 0.01 '*' 0.05 '.' 0.1 ' ' 1

*#aETR*

AnovaaETR<-**aov**(log_aETR**~**Temperatura**+**Tiempo**+**Temperatura*****Tiempo,data=AnalisisO2)

**summary**(AnovaaETR)

## Df Sum Sq Mean Sq F value Pr(>F)

| ## Temperatura | 1 | 0.03206 | 0.03206 | 5.655 0.0240 | * |
| --- | --- | --- | --- | --- | --- |
| ## Tiempo | 2 | 0.03638 | 0.01819 | 3.209 0.0546 | . |
| ## Temperatura:Tiempo | 2 | 0.02081 | 0.01040 | 1.835 0.1770 |  |
| ## Residuals | 30 | 0.17007 | 0.00567 |  |  |
| ## --- |  |  |  |  |  |

## Signif. codes: 0 '***' 0.001 '**' 0.01 '*' 0.05 '.' 0.1 ' ' 1

AnovaaETR2<-**aov**(sqrt_aETR**~**Temperatura**+**Tiempo**+**Temperatura*****Tiempo,data=AnalisisO2)

**summary**(AnovaaETR2)

| ## | Df | Sum Sq | Mean Sq F value Pr(>F) |
| --- | --- | --- | --- |
| ## Temperatura | 1 | 0.02137 | 0.021371 6.032 0.0201 * |
| ## Tiempo | 2 | 0.02287 | 0.011436 3.228 0.0537 . |
| ## Temperatura:Tiempo | 2 | 0.01391 | 0.006953 1.963 0.1581 |
| ## Residuals | 30 | 0.10628 | 0.003543 |
| ## --- |  |  |  |

## Signif. codes: 0 '***' 0.001 '**' 0.01 '*' 0.05 '.' 0.1 ' ' 1

*#ETR*

AnovaETR<-**aov**(log_ETR**~**Temperatura**+**Tiempo**+**Temperatura*****Tiempo,data=AnalisisO2)

**summary**(AnovaETR)

| ## | Df | Sum Sq | Mean Sq F | | value | Pr(>F) |
| --- | --- | --- | --- | --- | --- | --- |
| ## Temperatura | 1 | 0.00241 | 0.00241 | | 0.301 | 0.587212 |
| ## Tiempo | 2 | 0.05865 | 0.02932 | | 3.661 | 0.037772 * |
| ## Temperatura:Tiempo | 2 | 0.14143 | 0.07071 | | 8.830 | 0.000965 *** |
| ## Residuals | 30 | 0.24026 | 0.00801 | |  |  |
| ## --- |  |  |  | |  |  |
| ## Signif. codes: 0 '***' 0.001 | | | '**' 0.01 | | '*' 0.05 '.' 0.1 ' ' 1 | |
| AnovaETR2<-**aov**(sqrt_ETR**~**Temperatura**+**Tiempo**+**Temperatura*****Tiempo,data=AnalisisO2)  **summary**(AnovaETR2) | | | | | | |
| ## | Df | Sum Sq | Mean Sq | F value | | Pr(>F) |
| ## Temperatura | 1 | 0.055 | 0.055 | 0.115 | | 0.736832 |
| ## Tiempo | 2 | 4.126 | 2.063 | 4.292 | | 0.022939 * |
| ## Temperatura:Tiempo | 2 | 9.693 | 4.846 | 10.084 | | 0.000447 *** |
| ## Residuals | 30 | 14.417 | 0.481 |  | |  |
| ## --- |  |  |  |  | |  |
| ## Signif. codes: 0 '***' 0.001 | | | '**' 0.01 '*' 0.05 '.' 0.1 ' ' 1 | | | |
| *#Ek*  AnovaEk<-**aov**(log_Ek**~**Temperatura**+**Tiempo**+**Temperatura*****Tiempo,data=AnalisisO2)  **summary**(AnovaEk) | | | | | | |
| ## | Df | Sum Sq | Mean Sq F | | value Pr(>F) | |
| ## Temperatura | 1 | 0.0012 | 0.00120 | | 0.045 0.834 | |
| ## Tiempo | 2 | 0.0633 | 0.03167 | | 1.179 0.321 | |
| ## Temperatura:Tiempo | 2 | 0.0952 | 0.04758 | | 1.772 0.187 | |
| ## Residuals | 30 | 0.8055 | 0.02685 | |  | |
| AnovaEk2<-**aov**(sqrt_Ek**~**Temperatura**+**Tiempo**+**Temperatura*****Tiempo,data=AnalisisO2)  **summary**(AnovaEk2) | | | | | | |
| ## | Df | Sum Sq | Mean Sq F value Pr(>F) | | | |
| ## Temperatura | 1 | 0.08 | 0.080 0.020 0.889 | | | |
| ## Tiempo | 2 | 9.95 | 4.975 1.228 0.307 | | | |
| ## Temperatura:Tiempo | 2 | 13.55 | 6.776 1.672 0.205 | | | |
| ## Residuals | 30 | 121.58 | 4.053 | | | |
| *#fvfm*  Anovafvfm<-**aov**(log_fvfm**~**Temperatura**+**Tiempo**+**Temperatura*****Tiempo,data=AnalisisO2)  **summary**(Anovafvfm) | | | | | | |

## Df Sum Sq Mean Sq F value Pr(>F)

| ## Temperatura | 1 | 0.02048 | | 0.020477 18.864 0.000148 *** | | |
| --- | --- | --- | --- | --- | --- | --- |
| ## Tiempo | 2 | 0.01108 | | 0.005539 5.103 0.012371 * | | |
| ## Temperatura:Tiempo | 2 | 0.00325 | | 0.001623 1.495 0.240405 | | |
| ## Residuals | 30 | 0.03256 | | 0.001085 | | |
| ## --- |  |  | |  | | |
| ## Signif. codes: 0 '***' 0.001 | | | | '**' 0.01 '*' 0.05 '.' 0.1 ' ' 1 | | |
| Anovafvfm2<-**aov**(sqrt_fvfm**~**Temperatura**+**Tiempo**+**Temperatura*****Tiempo,data=AnalisisO2)  **summary**(Anovafvfm2) | | | | | | |
| ## | Df | Sum Sq | | Mean Sq F value Pr(>F) | | |
| ## Temperatura | 1 | 0.017999 | | 0.017999 19.073 | 0.000138 | *** |
| ## Tiempo | 2 | 0.009348 | | 0.004674 4.953 | 0.013843 | * |
| ## Temperatura:Tiempo | 2 | 0.002764 | | 0.001382 1.465 | 0.247199 |  |
| ## Residuals | 30 | 0.028310 | | 0.000944 |  |  |
| ## --- |  |  | |  |  |  |
| ## Signif. codes: 0 '***' 0.001 '**' 0.01 '*' 0.05 '.' 0.1 | | | | | | ' ' 1 |
| *#NPQ*  AnovaNPQ<-**aov**(log_NPQ**~**Temperatura**+**Tiempo**+**Temperatura*****Tiempo,data=AnalisisO2)  **summary**(AnovaNPQ) | | | | | | |
| ## | Df | Sum Sq | | Mean Sq F value Pr(>F) | | |
| ## Temperatura | 1 | 0.24140 | | 0.24140 25.764 1.88e-05 *** | | |
| ## Tiempo | 2 | 0.08487 | | 0.04244 4.529 0.0191 * | | |
| ## Temperatura:Tiempo | 2 | 0.00749 | | 0.00374 0.399 0.6742 | | |
| ## Residuals | 30 | 0.28108 | | 0.00937 | | |
| ## --- |  |  | |  | | |
| ## Signif. codes: 0 '***' 0.001 | | | | '**' 0.01 '*' 0.05 '.' 0.1 ' ' 1 | | |
| AnovaNPQ2<-**aov**(sqrt_NPQ**~**Temperatura**+**Tiempo**+**Temperatura*****Tiempo,data=AnalisisO2)  **summary**(AnovaNPQ2) | | | | | | |
| ## | Df | Sum Sq | Mean Sq F value Pr(>F) | | | |
| ## Temperatura | 1 | 0.7204 | 0.7204 25.779 1.87e-05 *** | | | |
| ## Tiempo | 2 | 0.2664 | 0.1332 4.766 0.0159 * | | | |
| ## Temperatura:Tiempo | 2 | 0.0129 | 0.0065 0.231 0.7949 | | | |
| ## Residuals | 30 | 0.8384 | 0.0279 | | | |
| ## --- |  |  |  | | | |
| ## Signif. codes: 0 '***' 0.001 | | | '**' 0.01 '*' 0.05 '.' 0.1 ' ' 1 | | | |

**TukeyHSD**(AnovaChla)

| ## | Tukey multiple comparisons of means | |
| --- | --- | --- |
| ## | 95% family-wise confidence level | |
| ## |  | |
| ## | Fit: aov(formula = log_Chla ~ Temperatura + Tiempo + Temperatura * Tiempo, data = AnalisisO2) | |
| ## |  | |
| ## | $Temperatura | |
| ## | diff lwr upr p adj | |
| ## | 18°C-15°C -0.008273003 -0.04469812 0.02815212 0.6461047 | |
| ## |  | |
| ## | $Tiempo | |
| ## | diff lwr upr p adj | |
| ## | 2-1 0.01243673 -0.04141475 | 0.066288212 0.8373357 |
| ## | 3-1 -0.06355843 -0.11740991 | -0.009706943 0.0180139 |
| ## | 3-2 -0.07599515 -0.12984664 | -0.022143672 0.0043266 |

##

| ## ## | $`Temperatura:Tiempo`  diff | lwr | upr | p adj |
| --- | --- | --- | --- | --- |
| ## | 18°C:1-15°C:1 -0.004081279 | -0.09804262 | 0.089880067 | 0.9999937 |
| ## | 15°C:2-15°C:1 0.028828050 | -0.06513330 | 0.122789395 | 0.9346303 |
| ## | 18°C:2-15°C:1 -0.008035871 | -0.10199722 | 0.085925475 | 0.9998200 |
| ## | 15°C:3-15°C:1 -0.073662160 | -0.16762351 | 0.020299186 | 0.1938219 |
| ## | 18°C:3-15°C:1 -0.057535970 | -0.15149732 | 0.036425375 | 0.4435205 |
| ## | 15°C:2-18°C:1 0.032909329 | -0.06105202 | 0.126870674 | 0.8911156 |
| ## | 18°C:2-18°C:1 -0.003954592 | -0.09791594 | 0.090006754 | 0.9999946 |
| ## | 15°C:3-18°C:1 -0.069580881 | -0.16354223 | 0.024380465 | 0.2448027 |
| ## | 18°C:3-18°C:1 -0.053454691 | -0.14741604 | 0.040506654 | 0.5234961 |
| ## | 18°C:2-15°C:2 -0.036863920 | -0.13082527 | 0.057097426 | 0.8365223 |
| ## | 15°C:3-15°C:2 -0.102490209 | -0.19645155 | -0.008528863 | 0.0262214 |
| ## | 18°C:3-15°C:2 -0.086364020 | -0.18032537 | 0.007597326 | 0.0858567 |
| ## | 15°C:3-18°C:2 -0.065626289 | -0.15958763 | 0.028335057 | 0.3024416 |
| ## | 18°C:3-18°C:2 -0.049500100 | -0.14346145 | 0.044461246 | 0.6033944 |
| ## | 18°C:3-15°C:3 0.016126189 | -0.07783516 | 0.110087535 | 0.9948573 |

**TukeyHSD**(AnovaChlc)

## Tukey multiple comparisons of means ## 95% family-wise confidence level ##

## Fit: aov(formula = log_Chlc ~ Temperatura + Tiempo + Temperatura * Tiempo, data = AnalisisO2) ##

| ##  ## | $Temperatura | | diff | lwr | upr | p adj |
| --- | --- | --- | --- | --- | --- | --- |
| ## | 18°C-15°C -0.00327603 | | | -0.03847539 | 0.03192333 0.8505301 | |
| ## |  | | |  |  | |
| ## | $Tiempo |  |  | |  |  |
| ## |  | diff | lwr | | upr | p adj |

| ## | 2-1 -0.01358656 -0.06562586 | 0.03845273 | 0.7973408 | |
| --- | --- | --- | --- | --- |
| ## | 3-1 -0.06245915 -0.11449845 | -0.01041985 | 0.0159979 | |
| ##  ## | 3-2 -0.04887259 -0.10091189 | 0.00316671 | 0.0689626 | |
| ## | $`Temperatura:Tiempo` |  |  |  |
| ## | diff | lwr | upr | p adj |
| ## | 18°C:1-15°C:1 -0.007081465 | -0.09788087 | 0.08371794 | 0.9998857 |
| ## | 15°C:2-15°C:1 -0.011467247 | -0.10226665 | 0.07933216 | 0.9987993 |
| ## | 18°C:2-15°C:1 -0.022787347 | -0.11358675 | 0.06801206 | 0.9715929 |
| ## | 15°C:3-15°C:1 -0.070286624 | -0.16108603 | 0.02051278 | 0.2046579 |
| ## | 18°C:3-15°C:1 -0.061713149 | -0.15251255 | 0.02908626 | 0.3307060 |
| ## | 15°C:2-18°C:1 -0.004385782 | -0.09518519 | 0.08641362 | 0.9999893 |
| ## | 18°C:2-18°C:1 -0.015705882 | -0.10650529 | 0.07509352 | 0.9946666 |
| ## | 15°C:3-18°C:1 -0.063205159 | -0.15400456 | 0.02759425 | 0.3058844 |
| ## | 18°C:3-18°C:1 -0.054631684 | -0.14543109 | 0.03616772 | 0.4627575 |
| ## | 18°C:2-15°C:2 -0.011320100 | -0.10211951 | 0.07947930 | 0.9988717 |
| ## | 15°C:3-15°C:2 -0.058819377 | -0.14961878 | 0.03198003 | 0.3820404 |
| ## | 18°C:3-15°C:2 -0.050245901 | -0.14104531 | 0.04055350 | 0.5528589 |
| ## | 15°C:3-18°C:2 -0.047499277 | -0.13829868 | 0.04330013 | 0.6104105 |
| ## | 18°C:3-18°C:2 -0.038925801 | -0.12972521 | 0.05187360 | 0.7805705 |
| ## | 18°C:3-15°C:3 0.008573475 | -0.08222593 | 0.09937288 | 0.9997076 |

**TukeyHSD**(AnovaFX)

## Tukey multiple comparisons of means ## 95% family-wise confidence level ##

## Fit: aov(formula = log_FX ~ Temperatura + Tiempo + Temperatura * Tiempo, data = AnalisisO2) ##

| ##  ## | $Temperatura | | diff | lwr | upr | p adj |
| --- | --- | --- | --- | --- | --- | --- |
| ## | 18°C-15°C -0.02996414 | | | -0.06935399 | 0.009425702 0.1307739 | |
| ## |  | | |  |  | |
| ## | $Tiempo |  |  | |  |  |
| ## |  | diff | lwr | | upr | p adj |

| ## | 2-1 -0.03391575 -0.09215033 | 0.024318832 | | 0.3358500 | |
| --- | --- | --- | --- | --- | --- |
| ## | 3-1 -0.06526023 -0.12349481 | -0.007025652 | | 0.0255114 | |
| ##  ## | 3-2 -0.03134448 -0.08957906 | 0.026890095 | | 0.3916854 | |
| ## | $`Temperatura:Tiempo` |  |  | |  |
| ## | diff | lwr | upr | | p adj |
| ## | 18°C:1-15°C:1 -0.017682440 | -0.11929152 | 0.083926636 | | 0.9945142 |
| ## | 15°C:2-15°C:1 -0.032968449 | -0.13457753 | 0.068640626 | | 0.9185509 |
| ## | 18°C:2-15°C:1 -0.052545484 | -0.15415456 | 0.049063592 | | 0.6217793 |
| ## | 15°C:3-15°C:1 -0.047784973 | -0.14939405 | 0.053824103 | | 0.7088228 |
| ## | 18°C:3-15°C:1 -0.100417929 | -0.20202701 | 0.001191146 | | 0.0541937 |
| ## | 15°C:2-18°C:1 -0.015286010 | -0.11689509 | 0.086323066 | | 0.9972309 |
| ## | 18°C:2-18°C:1 -0.034863044 | -0.13647212 | 0.066746032 | | 0.8991764 |
| ## | 15°C:3-18°C:1 -0.030102533 | -0.13171161 | 0.071506543 | | 0.9431965 |
| ## | 18°C:3-18°C:1 -0.082735490 | -0.18434457 | 0.018873586 | | 0.1632826 |
| ## | 18°C:2-15°C:2 -0.019577035 | -0.12118611 | 0.082032041 | | 0.9912276 |
| ## | 15°C:3-15°C:2 -0.014816523 | -0.11642560 | 0.086792553 | | 0.9976118 |
| ## | 18°C:3-15°C:2 -0.067449480 | -0.16905856 | 0.034159596 | | 0.3557313 |
| ## | 15°C:3-18°C:2 0.004760511 | -0.09684856 | 0.106369587 | | 0.9999908 |
| ## | 18°C:3-18°C:2 -0.047872445 | -0.14948152 | 0.053736631 | | 0.7072697 |
| ## | 18°C:3-15°C:3 -0.052632957 | -0.15424203 | 0.048976119 | | 0.6201469 |

**TukeyHSD**(AnovaROS)

## Tukey multiple comparisons of means ## 95% family-wise confidence level ##

## Fit: aov(formula = log_ROS ~ Temperatura + Tiempo + Temperatura * Tiempo, data = AnalisisO2) ##

## $Temperatura

## diff lwr upr p adj ## 18°C-15°C 0.1389311 0.05126555 0.2265966 0.0029468 ##

## $Tiempo

## diff lwr upr p adj ## 2-1 -0.01970287 -0.14930899 0.1099032 0.9256921

## 3-1 0.06942556 -0.06018055 0.1990317 0.3950927

## 3-2 0.08912844 -0.04047767 0.2187345 0.2235146 ##

## $`Temperatura:Tiempo`

## diff lwr upr p adj

| ## | 18°C:1-15°C:1 | 0.1784303981 | -0.04770942 | 0.40457022 | 0.1884347 |
| --- | --- | --- | --- | --- | --- |
| ## | 15°C:2-15°C:1 | 0.0003550918 | -0.22578473 | 0.22649491 | 1.0000000 |
| ## | 18°C:2-15°C:1 | 0.1386695574 | -0.08747026 | 0.36480938 | 0.4419715 |
| ## | 15°C:3-15°C:1 | 0.1086165950 | -0.11752323 | 0.33475642 | 0.6906037 |
| ## | 18°C:3-15°C:1 | 0.2086649305 | -0.01747489 | 0.43480475 | 0.0838898 |
| ## | 15°C:2-18°C:1 | -0.1780753063 | -0.40421513 | 0.04806451 | 0.1900927 |
| ## | 18°C:2-18°C:1 | -0.0397608406 | -0.26590066 | 0.18637898 | 0.9942456 |
| ## | 15°C:3-18°C:1 | -0.0698138030 | -0.29595362 | 0.15632602 | 0.9329949 |
| ## | 18°C:3-18°C:1 | 0.0302345324 | -0.19590529 | 0.25637435 | 0.9984214 |
| ## | 18°C:2-15°C:2 | 0.1383144657 | -0.08782535 | 0.36445429 | 0.4447813 |
| ## | 15°C:3-15°C:2 | 0.1082615033 | -0.11787832 | 0.33440132 | 0.6934761 |
| ## | 18°C:3-15°C:2 | 0.2083098387 | -0.01782998 | 0.43444966 | 0.0847474 |
| ## | 15°C:3-18°C:2 | -0.0300529624 | -0.25619278 | 0.19608686 | 0.9984663 |
| ## | 18°C:3-18°C:2 | 0.0699953731 | -0.15614445 | 0.29613519 | 0.9323005 |
| ## | 18°C:3-15°C:3 | 0.1000483355 | -0.12609148 | 0.32618816 | 0.7576907 |

**TukeyHSD**(AnovaMDA)

## Tukey multiple comparisons of means ## 95% family-wise confidence level ##

## Fit: aov(formula = log_MDA ~ Temperatura + Tiempo + Temperatura * Tiempo, data = AnalisisO2) ##

| ## | $Temperatura |  | | | | | | |
| --- | --- | --- | --- | --- | --- | --- | --- | --- |
| ## |  | diff | lwr | | upr | | p adj | |
| ## | 18°C-15°C 0.01693149 | | -0.001456914 | | 0.0353199 0.0697852 | | | |
| ## |  | |  | |  | | | |
| ## | $Tiempo |  | |  | |  | | |
| ## | diff | lwr | | upr | | p adj | | |
| ## | 2-1 0.01261789 | -0.014567831 | | 0.03980361 | | 0.4950534 | | |
| ## | 3-1 0.03403239 | 0.006846672 | | 0.06121811 | | 0.0117199 | | |
| ## | 3-2 0.02141450 | -0.005771215 | | 0.04860022 | | 0.1445583 | | |
| ## |  |  | |  | |  | | |
| ## $`Temperatura:Tiempo` | | | | | | | | |
| ## |  | diff | | lwr | | upr | | p adj |
| ## | 18°C:1-15°C:1 | 0.018212476 | | -0.029221810 | | 0.06564676 | | 0.8483157 |
| ## | 15°C:2-15°C:1 | 0.013624894 | | -0.033809393 | | 0.06105918 | | 0.9499163 |
| ## | 18°C:2-15°C:1 | 0.029823357 | | -0.017610930 | | 0.07725764 | | 0.4145823 |
| ## | 15°C:3-15°C:1 | 0.034946855 | | -0.012487431 | | 0.08238114 | | 0.2496539 |
| ## | 18°C:3-15°C:1 | 0.051330401 | | 0.003896114 | | 0.09876469 | | 0.0279247 |
| ## | 15°C:2-18°C:1 | -0.004587583 | | -0.052021869 | | 0.04284670 | | 0.9996712 |
| ## | 18°C:2-18°C:1 | 0.011610880 | | -0.035823406 | | 0.05904517 | | 0.9744911 |
| ## | 15°C:3-18°C:1 | 0.016734379 | | -0.030699907 | | 0.06416867 | | 0.8881509 |
| ## | 18°C:3-18°C:1 | 0.033117924 | | -0.014316362 | | 0.08055221 | | 0.3028135 |
| ## | 18°C:2-15°C:2 | 0.016198463 | | -0.031235823 | | 0.06363275 | | 0.9009537 |
| ## | 15°C:3-15°C:2 | 0.021321962 | | -0.026112325 | | 0.06875625 | | 0.7455429 |
| ## | 18°C:3-15°C:2 | 0.037705507 | | -0.009728779 | | 0.08513979 | | 0.1823302 |
| ## | 15°C:3-18°C:2 | 0.005123499 | | -0.042310788 | | 0.05255779 | | 0.9994365 |
| ## | 18°C:3-18°C:2 | 0.021507044 | | -0.025927242 | | 0.06894133 | | 0.7387676 |
| ## | 18°C:3-15°C:3 | 0.016383545 | | -0.031050741 | | 0.06381783 | | 0.8966333 |
| **TukeyHSD**(AnovaFI) | | | | | | | | |

## Tukey multiple comparisons of means ## 95% family-wise confidence level

##

## Fit: aov(formula = log_FI ~ Temperatura + Tiempo + Temperatura * Tiempo, data = AnalisisO2) ##

## $Temperatura

## diff lwr upr p adj ## 18°C-15°C 0.07982156 0.005614101 0.154029 0.0359001 ##

| ## ## | $Tiempo  diff lwr upr | p adj |  |
| --- | --- | --- | --- |
| ## | 2-1 -0.03914056 -0.14885005 0.07056894 | 0.6571515 |  |
| ## | 3-1 -0.01904669 -0.12875618 0.09066280 | 0.9042826 |  |
| ## | 3-2 0.02009387 -0.08961562 0.12980336 | 0.8941038 |  |
| ## |  |  |  |
| ## | $`Temperatura:Tiempo` |  |  |
| ## | diff lwr | upr | p adj |
| ## | 18°C:1-15°C:1 0.35206826 0.16064453 | 0.54349199 | 0.0000596 |
| ## | 15°C:2-15°C:1 0.13531865 -0.05610508 | 0.32674238 | 0.2902011 |
| ## | 18°C:2-15°C:1 0.13846850 -0.05295523 | 0.32989223 | 0.2673365 |
| ## | 15°C:3-15°C:1 0.21486416 0.02344043 | 0.40628789 | 0.0207610 |
| ## | 18°C:3-15°C:1 0.09911072 -0.09231301 | 0.29053445 | 0.6206001 |
| ## | 15°C:2-18°C:1 -0.21674962 -0.40817335 | -0.02532589 | 0.0192923 |
| ## | 18°C:2-18°C:1 -0.21359977 -0.40502350 | -0.02217604 | 0.0218036 |
| ## | 15°C:3-18°C:1 -0.13720410 -0.32862783 | 0.05421963 | 0.2763671 |
| ## | 18°C:3-18°C:1 -0.25295754 -0.44438127 | -0.06153381 | 0.0044444 |
| ## | 18°C:2-15°C:2 0.00314985 -0.18827388 | 0.19457358 | 1.0000000 |
| ## | 15°C:3-15°C:2 0.07954551 -0.11187822 | 0.27096924 | 0.8016478 |
| ## | 18°C:3-15°C:2 -0.03620793 -0.22763166 | 0.15521581 | 0.9919379 |
| ## | 15°C:3-18°C:2 0.07639566 -0.11502807 | 0.26781939 | 0.8266907 |
| ## | 18°C:3-18°C:2 -0.03935778 -0.23078151 | 0.15206595 | 0.9882133 |
| ## | 18°C:3-15°C:3 -0.11575344 -0.30717717 | 0.07567029 | 0.4572727 |

**TukeyHSD**(AnovaFS)

## Tukey multiple comparisons of means ## 95% family-wise confidence level ##

## Fit: aov(formula = log_FS ~ Temperatura + Tiempo + Temperatura * Tiempo, data = AnalisisO2) ##

## $Temperatura

## diff lwr upr p adj ## 18°C-15°C 0.01562995 -0.02464153 0.05590143 0.434214 ##

| ##  ## | $Tiempo | diff | lwr | upr | p adj | |
| --- | --- | --- | --- | --- | --- | --- |
| ## | 2-1 -0.07036031 -0.1298983 | | | -0.01082230 | 0.0178529 | |
| ## | 3-1 -0.15172839 -0.2112664 | | | -0.09219039 | 0.0000019 | |
| ## | 3-2 -0.08136809 -0.1409061 | | | -0.02183009 | 0.0057446 | |
| ## |  |  |  |  |  | |
| ## | $`Temperatura:Tiempo` | | |  |  |  |
| ## | diff | | | lwr | upr | p adj |
| ## | 18°C:1-15°C:1 0.08724796 | | | -0.01663536 | 0.191131282 | 0.1405136 |
| ## | 15°C:2-15°C:1 -0.07182933 | | | -0.17571265 | 0.032053992 | 0.3127962 |
| ## | 18°C:2-15°C:1 0.01835668 | | | -0.08552664 | 0.122239998 | 0.9941107 |
| ## | 15°C:3-15°C:1 -0.04283235 | | | -0.14671567 | 0.061050969 | 0.8066909 |
| ## | 18°C:3-15°C:1 -0.17337648 | | | -0.27725979 | -0.069493156 | 0.0002512 |

| ## | 15°C:2-18°C:1 | -0.15907729 | -0.26296061 | -0.055193972 | 0.0007967 |
| --- | --- | --- | --- | --- | --- |
| ## | 18°C:2-18°C:1 | -0.06889128 | -0.17277460 | 0.034992034 | 0.3567847 |
| ## | 15°C:3-18°C:1 | -0.13008031 | -0.23396363 | -0.026196995 | 0.0076976 |
| ## | 18°C:3-18°C:1 | -0.26062444 | -0.36450776 | -0.156741120 | 0.0000002 |
| ## | 18°C:2-15°C:2 | 0.09018601 | -0.01369731 | 0.194069325 | 0.1184171 |
| ## | 15°C:3-15°C:2 | 0.02899698 | -0.07488634 | 0.132880296 | 0.9554895 |
| ## | 18°C:3-15°C:2 | -0.10154715 | -0.20543047 | 0.002336171 | 0.0583181 |
| ## | 15°C:3-18°C:2 | -0.06118903 | -0.16507235 | 0.042694290 | 0.4859613 |
| ## | 18°C:3-18°C:2 | -0.19173315 | -0.29561647 | -0.087849835 | 0.0000565 |
| ## | 18°C:3-15°C:3 | -0.13054413 | -0.23442744 | -0.026660807 | 0.0074325 |

**TukeyHSD**(AnovaDPPH)

## Tukey multiple comparisons of means ## 95% family-wise confidence level ##

## Fit: aov(formula = log_DPPH ~ Temperatura + Tiempo + Temperatura * Tiempo, data = AnalisisO2) ##

| ##  ## | $Temperatura | | diff | lwr | upr | p adj |
| --- | --- | --- | --- | --- | --- | --- |
| ## | 18°C-15°C -0.03127697 | | | -0.06388397 | 0.001330035 0.0594685 | |
| ## |  | | |  |  | |
| ## | $Tiempo |  |  | |  |  |
| ## |  | diff | lwr | | upr | p adj |

| ## | 2-1 -0.04044265 -0.08864936 | 0.007764068 | | 0.1136961 | |
| --- | --- | --- | --- | --- | --- |
| ## | 3-1 -0.09865216 -0.14685887 | -0.050445440 | | 0.0000596 | |
| ##  ## | 3-2 -0.05820951 -0.10641622 | -0.010002794 | | 0.0153181 | |
| ## | $`Temperatura:Tiempo` |  |  | |  |
| ## | diff | lwr | upr | | p adj |
| ## | 18°C:1-15°C:1 -0.001886161 | -0.08599838 | 0.082226060 | | 0.9999998 |
| ## | 15°C:2-15°C:1 -0.038709945 | -0.12282217 | 0.045402277 | | 0.7268022 |
| ## | 18°C:2-15°C:1 -0.044061511 | -0.12817373 | 0.040050711 | | 0.6090445 |
| ## | 15°C:3-15°C:1 -0.056298649 | -0.14041087 | 0.027813572 | | 0.3469084 |
| ## | 18°C:3-15°C:1 -0.142891824 | -0.22700405 | -0.058779603 | | 0.0001953 |
| ## | 15°C:2-18°C:1 -0.036823783 | -0.12093600 | 0.047288438 | | 0.7655009 |
| ## | 18°C:2-18°C:1 -0.042175349 | -0.12628757 | 0.041936872 | | 0.6514341 |
| ## | 15°C:3-18°C:1 -0.054412488 | -0.13852471 | 0.029699734 | | 0.3835328 |
| ## | 18°C:3-18°C:1 -0.141005663 | -0.22511788 | -0.056893441 | | 0.0002359 |
| ## | 18°C:2-15°C:2 -0.005351566 | -0.08946379 | 0.078760655 | | 0.9999582 |
| ## | 15°C:3-15°C:2 -0.017588704 | -0.10170093 | 0.066523517 | | 0.9872801 |
| ## | 18°C:3-15°C:2 -0.104181879 | -0.18829410 | -0.020069658 | | 0.0085600 |
| ## | 15°C:3-18°C:2 -0.012237138 | -0.09634936 | 0.071875083 | | 0.9976376 |
| ## | 18°C:3-18°C:2 -0.098830313 | -0.18294253 | -0.014718092 | | 0.0139827 |
| ## | 18°C:3-15°C:3 -0.086593175 | -0.17070540 | -0.002480954 | | 0.0407066 |

**TukeyHSD**(AnovaaETR)

## Tukey multiple comparisons of means ## 95% family-wise confidence level ##

## Fit: aov(formula = log_aETR ~ Temperatura + Tiempo + Temperatura * Tiempo, data = AnalisisO2) ##

## $Temperatura

## diff lwr upr p adj

| ## | 18°C-15°C -0.05968484 -0.1109414 -0.008428308 0.0239721 | | | | | | |
| --- | --- | --- | --- | --- | --- | --- | --- |
| ## |  | | | | | | |
| ## | $Tiempo |  |  |  | |  | |
| ## |  | diff | lwr | upr | | p adj | |
| ## | 2-1 0.04334802 -0.03243045 | | | 0.119126490 | | 0.3485178 | |
| ## | 3-1 -0.03434879 -0.11012726 | | | 0.041429680 | | 0.5110054 | |
| ## | 3-2 -0.07769681 -0.15347528 | | | -0.001918337 | | 0.0435791 | |
| ## |  |  |  |  | |  | |
| ## | $`Temperatura:Tiempo` | | |  |  | |  |
| ## | diff | | | lwr | upr | | p adj |
| ## | 18°C:1-15°C:1 0.008135487 | | | -0.12408459 | 0.14035557 | | 0.9999646 |
| ## | 15°C:2-15°C:1 0.096356328 | | | -0.03586375 | 0.22857641 | | 0.2601072 |
| ## | 18°C:2-15°C:1 -0.001524806 | | | -0.13374488 | 0.13069527 | | 1.0000000 |
| ## | 15°C:3-15°C:1 0.014373382 | | | -0.11784670 | 0.14659346 | | 0.9994186 |
| ## | 18°C:3-15°C:1 -0.074935479 | | | -0.20715556 | 0.05728460 | | 0.5275507 |
| ## | 15°C:2-18°C:1 0.088220841 | | | -0.04399924 | 0.22044092 | | 0.3502548 |
| ## | 18°C:2-18°C:1 -0.009660293 | | | -0.14188037 | 0.12255978 | | 0.9999171 |
| ## | 15°C:3-18°C:1 0.006237895 | | | -0.12598218 | 0.13845797 | | 0.9999905 |
| ## | 18°C:3-18°C:1 -0.083070967 | | | -0.21529104 | 0.04914911 | | 0.4153689 |
| ## | 18°C:2-15°C:2 -0.097881134 | | | -0.23010121 | 0.03433894 | | 0.2451048 |
| ## | 15°C:3-15°C:2 -0.081982946 | | | -0.21420302 | 0.05023713 | | 0.4298038 |
| ## | 18°C:3-15°C:2 -0.171291807 | | | -0.30351189 | -0.03907173 | | 0.0054669 |
| ## | 15°C:3-18°C:2 0.015898188 | | | -0.11632189 | 0.14811827 | | 0.9990524 |
| ## | 18°C:3-18°C:2 -0.073410674 | | | -0.20563075 | 0.05880940 | | 0.5493615 |
| ## | 18°C:3-15°C:3 -0.089308862 | | | -0.22152894 | 0.04291122 | | 0.3372490 |

**TukeyHSD**(AnovaETR)

## Tukey multiple comparisons of means ## 95% family-wise confidence level ##

## Fit: aov(formula = log_ETR ~ Temperatura + Tiempo + Temperatura * Tiempo, data = AnalisisO2) ##

## $Temperatura

## diff lwr upr p adj ## 18°C-15°C 0.01637058 -0.04455112 0.07729228 0.5872116 ##

| ## | $Tiempo |  | | | | | |
| --- | --- | --- | --- | --- | --- | --- | --- |
| ## |  | diff | lwr | upr | p | adj | |
| ## | 2-1 0.04106300 -0.049004617 0.1311306 | | | | 0.5071204 | |  |
| ## | 3-1 0.09841774 0.008350119 0.1884854 | | | | 0.0299291 | |  |
| ## | 3-2 0.05735474 -0.032712882 0.1474224 | | | | 0.2740212 | |  |
| ## |  | | | |  | |  |
| ## | $`Temperatura:Tiempo` | | | |  | |  |
| ## | diff lwr | | | | upr | | p adj |
| ## | 18°C:1-15°C:1 0.08941123 -0.06774089 | | | | 0.246563345 | | 0.5234093 |
| ## | 15°C:2-15°C:1 0.02589773 -0.13125439 | | | | 0.183049848 | | 0.9957461 |
| ## | 18°C:2-15°C:1 0.14563950 -0.01151262 | | | | 0.302791618 | | 0.0817299 |
| ## | 15°C:3-15°C:1 0.22314398 0.06599186 | | | | 0.380296102 | | 0.0020007 |
| ## | 18°C:3-15°C:1 0.06310272 -0.09404940 | | | | 0.220254837 | | 0.8230648 |
| ## | 15°C:2-18°C:1 -0.06351350 -0.22066562 | | | | 0.093638622 | | 0.8191530 |
| ## | 18°C:2-18°C:1 0.05622827 -0.10092385 | | | | 0.213380392 | | 0.8821959 |
| ## | 15°C:3-18°C:1 0.13373276 -0.02341936 | | | | 0.290884876 | | 0.1314634 |
| ## | 18°C:3-18°C:1 -0.02630851 -0.18346063 | | | | 0.130843611 | | 0.9954211 |
| ## | 18°C:2-15°C:2 0.11974177 -0.03741035 | | | | 0.276893889 | | 0.2185826 |

## 15°C:3-15°C:2 0.19724625 0.04009414 0.354398373 0.0075213

## 18°C:3-15°C:2 0.03720499 -0.11994713 0.194357108 0.9779365

## 15°C:3-18°C:2 0.07750448 -0.07964763 0.234656603 0.6668268

## 18°C:3-18°C:2 -0.08253678 -0.23968890 0.074615338 0.6064569

## 18°C:3-15°C:3 -0.16004127 -0.31719338 -0.002889146 0.0440068

**TukeyHSD**(AnovaEk)

## Tukey multiple comparisons of means ## 95% family-wise confidence level ##

## Fit: aov(formula = log_Ek ~ Temperatura + Tiempo + Temperatura * Tiempo, data = AnalisisO2) ##

## $Temperatura

## diff lwr upr p adj ## 18°C-15°C -0.01157028 -0.1231222 0.09998164 0.8336749

| ## |  |  |  |  |  |  |  |  |
| --- | --- | --- | --- | --- | --- | --- | --- | --- |
| ## | $Tiempo |  |  |  |  |  |  |  |
| ## |  | diff | | lwr | upr | p | adj | |
| ## | 2-1 -0.06396853 | | | -0.22888868 | 0.1009516 | 0.6096539 | | |
| ## | 3-1 0.03764114 | | | -0.12727901 | 0.2025613 | 0.8407841 | | |
| ## | 3-2 0.10160967 | | | -0.06331048 | 0.2665298 | 0.2965869 | | |
| ## |  | | |  |  |  | | |
| ## $`Temperatura:Tiempo` | | | | | | | | |
| ## |  | | diff | | lwr | upr | | p adj |
| ## | 18°C:1-15°C:1 | | 0.034711305 | | -0.25304529 | 0.3224679 | | 0.9990376 |
| ## | 15°C:2-15°C:1 | | -0.088949438 | | -0.37670603 | 0.1988072 | | 0.9326548 |
| ## | 18°C:2-15°C:1 | | -0.004276314 | | -0.29203291 | 0.2834803 | | 1.0000000 |
| ## | 15°C:3-15°C:1 | | 0.132044424 | | -0.15571217 | 0.4198010 | | 0.7291742 |
| ## | 18°C:3-15°C:1 | | -0.022050840 | | -0.30980743 | 0.2657058 | | 0.9998952 |
| ## | 15°C:2-18°C:1 | | -0.123660743 | | -0.41141733 | 0.1640958 | | 0.7788672 |
| ## | 18°C:2-18°C:1 | | -0.038987619 | | -0.32674421 | 0.2487690 | | 0.9983176 |
| ## | 15°C:3-18°C:1 | | 0.097333119 | | -0.19042347 | 0.3850897 | | 0.9044648 |
| ## | 18°C:3-18°C:1 | | -0.056762145 | | -0.34451874 | 0.2309944 | | 0.9902332 |
| ## | 18°C:2-15°C:2 | | 0.084673124 | | -0.20308347 | 0.3724297 | | 0.9447384 |
| ## | 15°C:3-15°C:2 | | 0.220993862 | | -0.06676273 | 0.5087505 | | 0.2115692 |
| ## | 18°C:3-15°C:2 | | 0.066898598 | | -0.22085799 | 0.3546552 | | 0.9796238 |
| ## | 15°C:3-18°C:2 | | 0.136320738 | | -0.15143585 | 0.4240773 | | 0.7025785 |
| ## | 18°C:3-18°C:2 | | -0.017774526 | | -0.30553112 | 0.2699821 | | 0.9999639 |
| ## | 18°C:3-15°C:3 | | -0.154095265 | | -0.44185186 | 0.1336613 | | 0.5868488 |

**TukeyHSD**(Anovafvfm)

## Tukey multiple comparisons of means ## 95% family-wise confidence level ##

## Fit: aov(formula = log_fvfm ~ Temperatura + Tiempo + Temperatura * Tiempo, data = AnalisisO2) ##

| ##  ## | $Temperatura | | diff | lwr | upr | p adj |
| --- | --- | --- | --- | --- | --- | --- |
| ## | 18°C-15°C -0.04769878 | | | -0.07012725 | -0.02527032 0.0001475 | |
| ## |  | | |  |  | |
| ## | $Tiempo |  |  | |  |  |
| ## |  | diff | lwr | | upr | p adj |
| ## | 2-1 -0.02864758 -0.06180619 0.004511018 0.1007216 | | | | | |

| ## | 3-1 -0.04206031 -0.07521891 | -0.008901708 | 0.0105893 | |
| --- | --- | --- | --- | --- |
| ##  ## | 3-2 -0.01341273 -0.04657133 | 0.019745877 | 0.5842982 | |
| ## | $`Temperatura:Tiempo` |  |  |  |
| ## | diff | lwr | upr | p adj |
| ## | 18°C:1-15°C:1 -0.0208806671 | -0.07873658 | 0.036975250 | 0.8784047 |
| ## | 15°C:2-15°C:1 -0.0091747502 | -0.06703067 | 0.048681167 | 0.9964487 |
| ## | 18°C:2-15°C:1 -0.0690010868 | -0.12685700 | -0.011145169 | 0.0122177 |
| ## | 15°C:3-15°C:1 -0.0213059709 | -0.07916189 | 0.036549947 | 0.8691898 |
| ## | 18°C:3-15°C:1 -0.0836953170 | -0.14155123 | -0.025839400 | 0.0016069 |
| ## | 15°C:2-18°C:1 0.0117059169 | -0.04615000 | 0.069561834 | 0.9890396 |
| ## | 18°C:2-18°C:1 -0.0481204196 | -0.10597634 | 0.009735498 | 0.1474537 |
| ## | 15°C:3-18°C:1 -0.0004253037 | -0.05828122 | 0.057430614 | 1.0000000 |
| ## | 18°C:3-18°C:1 -0.0628146499 | -0.12067057 | -0.004958732 | 0.0272080 |
| ## | 18°C:2-15°C:2 -0.0598263365 | -0.11768225 | -0.001970419 | 0.0394179 |
| ## | 15°C:3-15°C:2 -0.0121312206 | -0.06998714 | 0.045724697 | 0.9871234 |
| ## | 18°C:3-15°C:2 -0.0745205668 | -0.13237648 | -0.016664649 | 0.0058010 |
| ## | 15°C:3-18°C:2 0.0476951159 | -0.01016080 | 0.105551033 | 0.1539568 |
| ## | 18°C:3-18°C:2 -0.0146942302 | -0.07255015 | 0.043161687 | 0.9700997 |
| ## | 18°C:3-15°C:3 -0.0623893461 | -0.12024526 | -0.004533429 | 0.0287021 |

**TukeyHSD**(AnovaNPQ)

## Tukey multiple comparisons of means ## 95% family-wise confidence level ##

## Fit: aov(formula = log_NPQ ~ Temperatura + Tiempo + Temperatura * Tiempo, data = AnalisisO2) ##

## $Temperatura

## diff lwr upr p adj ## 18°C-15°C 0.1637737 0.09787917 0.2296683 1.88e-05 ##

| ## ## | $Tiempo  diff | lwr | | upr | p adj | |
| --- | --- | --- | --- | --- | --- | --- |
| ## | 2-1 0.02363361 | -0.073785959 | | 0.1210532 | 0.8222085 | |
| ## | 3-1 0.11276325 | 0.015343689 | | 0.2101828 | 0.0205957 | |
| ## | 3-2 0.08912965 | -0.008289916 | | 0.1865492 | 0.0781015 | |
| ## |  |  | |  |  | |
| ## | $`Temperatura:Tiempo` | |  | |  |  |
| ## | diff | | lwr | | upr | p adj |
| ## | 18°C:1-15°C:1 0.18896874 | | 0.018988769 | | 0.358948705 | 0.0224810 |
| ## | 15°C:2-15°C:1 0.02864219 | | -0.141337777 | | 0.198622159 | 0.9952794 |
| ## | 18°C:2-15°C:1 0.20759376 | | 0.037613788 | | 0.377573724 | 0.0097952 |
| ## | 15°C:3-15°C:1 0.14554719 | | -0.024432776 | | 0.315527160 | 0.1273288 |
| ## | 18°C:3-15°C:1 0.26894805 | | 0.098968082 | | 0.438928018 | 0.0005206 |
| ## | 15°C:2-18°C:1 -0.16032655 | | -0.330306514 | | 0.009653422 | 0.0733705 |
| ## | 18°C:2-18°C:1 0.01862502 | | -0.151354949 | | 0.188604987 | 0.9993958 |
| ## | 15°C:3-18°C:1 -0.04342154 | | -0.213401513 | | 0.126558423 | 0.9693520 |
| ## | 18°C:3-18°C:1 0.07997931 | | -0.090000655 | | 0.249959281 | 0.7083910 |
| ## | 18°C:2-15°C:2 0.17895156 | | 0.008971597 | | 0.348931533 | 0.0345079 |
| ## | 15°C:3-15°C:2 0.11690500 | | -0.053074967 | | 0.286884969 | 0.3183354 |
| ## | 18°C:3-15°C:2 0.24030586 | | 0.070325891 | | 0.410285827 | 0.0021048 |
| ## | 15°C:3-18°C:2 -0.06204656 | | -0.232026532 | | 0.107933404 | 0.8732918 |
| ## | 18°C:3-18°C:2 0.06135429 | | -0.108625674 | | 0.231334262 | 0.8783530 |
| ## | 18°C:3-15°C:3 0.12340086 | | -0.046579110 | | 0.293380826 | 0.2638207 |

**install.packages**("foreign")

## Installing package into '/cloud/lib/x86_64-pc-linux-gnu-library/4.3' ## (as 'lib' is unspecified)

**install.packages**("agricolae")

## Installing package into '/cloud/lib/x86_64-pc-linux-gnu-library/4.3' ## (as 'lib' is unspecified)

**library**(foreign) **library**(agricolae)

tx <- **with**(AnalisisO2, **interaction**(Temperatura, Tiempo)) modelo <- **aov**(sqrt_Chla**~** tx, data=AnalisisO2)

out<-**HSD.test**(modelo, "tx", group=TRUE, alpha=0.05) out

| ## | $statistics | |  | | | | | | | |
| --- | --- | --- | --- | --- | --- | --- | --- | --- | --- | --- |
| ## | MSerror Df | | Mean | CV | | MSD | | | | |
| ## | 0.002777837 30 0.8668297 6.080225 | | | | | 0.09255376 | |  |  |  |
| ## |  | | | | |  | |  |  |  |
| ## | $parameters | | | | |  | |  |  |  |
| ## | test name.t ntr StudentizedRange | | | | | alpha | |  |  |  |
| ## | Tukey tx 6 4.301464 | | | | | 0.05 | |  |  |  |
| ## |  | | | | |  | |  |  |  |
| ## | $means | | | | |  | |  |  |  |
| ## | sqrt_Chla std r | | | | | se | | Min | Max | Q25 |
| ## | 15°C.1 | 0.8845815 | 0.03613801 | 6 | 0.02151681 | | 0.8358978 | | 0.9444413 | 0.8692961 |
| ## | 15°C.2 | 0.9144307 | 0.03715154 | 6 | 0.02151681 | | 0.8641749 | | 0.9696120 | 0.8919082 |
| ## | 15°C.3 | 0.8136426 | 0.05383004 | 6 | 0.02151681 | | 0.7176780 | | 0.8701541 | 0.8002115 |
| ## | 18°C.1 | 0.8827649 | 0.07864180 | 6 | 0.02151681 | | 0.7899539 | | 0.9788387 | 0.8154537 |
| ## | 18°C.2 | 0.8775332 | 0.05893190 | 6 | 0.02151681 | | 0.7814086 | | 0.9283144 | 0.8464208 |
| ## | 18°C.3 | 0.8280251 | 0.03775788 | 6 | 0.02151681 | | 0.7889243 | | 0.8837765 | 0.7973214 |
| ## |  | Q50 | Q75 |  |  | |  | |  |  |
| ## | 15°C.1 | 0.8819676 | 0.8940988 | | | | | | | |
| ## | 15°C.2 | 0.9154058 | 0.9322840 | | | | | | | |
| ## | 15°C.3 | 0.8266684 | 0.8436380 | | | | | | | |
| ## | 18°C.1 | 0.8916281 | 0.9387657 | | | | | | | |
| ## | 18°C.2 | 0.8970278 | 0.9231587 | | | | | | | |
| ## | 18°C.3 | 0.8220830 | 0.8521828 | | | | | | | |
| ## |  |  |  | | | | | | | |
| ## | $comparison | |  | | | | | | | |
| ## | NULL | |  | | | | | | | |
| ## |  | |  | | | | | | | |
| ## | $groups | |  | | | | | | | |
| ## | sqrt_Chla | | groups | | | | | | | |
| ## | 15°C.2 0.9144307 | | a | | | | | | | |
| ## | 15°C.1 0.8845815 | | ab | | | | | | | |
| ## | 18°C.1 0.8827649 | | ab | | | | | | | |
| ## | 18°C.2 0.8775332 | | ab | | | | | | | |
| ## | 18°C.3 0.8280251 | | ab | | | | | | | |
| ## | 15°C.3 0.8136426 | | b | | | | | | | |
| ## |  | |  | | | | | | | |
| ## | attr(,"class") | |  | | | | | | | |
| ## | [1] "group" | |  | | | | | | | |

tx <- **with**(AnalisisO2, **interaction**(Temperatura, Tiempo)) modelo <- **aov**(sqrt_Chlc**~** tx, data=AnalisisO2)

out<-**HSD.test**(modelo, "tx", group=TRUE, alpha=0.05) out

| ## | $statistics | |  | | | | | | | |
| --- | --- | --- | --- | --- | --- | --- | --- | --- | --- | --- |
| ## | MSerror Df | | Mean | CV | | MSD | | | | |
| ## | 0.001031305 30 0.5351044 6.001434 | | | | | 0.05639417 | |  |  |  |
| ## |  | | | | |  | |  |  |  |
| ## | $parameters | | | | |  | |  |  |  |
| ## | test name.t ntr StudentizedRange | | | | | alpha | |  |  |  |
| ## | Tukey tx 6 4.301464 | | | | | 0.05 | |  |  |  |
| ## |  | | | | |  | |  |  |  |
| ## | $means | | | | |  | |  |  |  |
| ## | sqrt_Chlc std r | | | | | se | | Min | Max | Q25 |
| ## | 15°C.1 | 0.5523801 | 0.01889833 | 6 | 0.01311046 | | 0.5314686 | | 0.5792747 | 0.5371593 |
| ## | 15°C.2 | 0.5463147 | 0.04375054 | 6 | 0.01311046 | | 0.4994403 | | 0.6135125 | 0.5109847 |
| ## | 15°C.3 | 0.5094000 | 0.01577913 | 6 | 0.01311046 | | 0.4859215 | | 0.5264484 | 0.4999999 |
| ## | 18°C.1 | 0.5484593 | 0.03328231 | 6 | 0.01311046 | | 0.5083650 | | 0.6031077 | 0.5271750 |
| ## | 18°C.2 | 0.5383654 | 0.02642958 | 6 | 0.01311046 | | 0.4964360 | | 0.5676987 | 0.5249483 |
| ## | 18°C.3 | 0.5157069 | 0.04314345 | 6 | 0.01311046 | | 0.4719261 | | 0.5862767 | 0.4854000 |
| ## |  | Q50 | Q75 |  |  | |  | |  |  |
| ## | 15°C.1 | 0.5505995 | 0.5648941 | | | | | | | |
| ## | 15°C.2 | 0.5465845 | 0.5661324 | | | | | | | |
| ## | 15°C.3 | 0.5118324 | 0.5211901 | | | | | | | |
| ## | 18°C.1 | 0.5524872 | 0.5548000 | | | | | | | |
| ## | 18°C.2 | 0.5435998 | 0.5559950 | | | | | | | |
| ## | 18°C.3 | 0.5050235 | 0.5366052 | | | | | | | |
| ## |  |  |  | | | | | | | |
| ## | $comparison | |  | | | | | | | |
| ## | NULL | |  | | | | | | | |
| ## |  | |  | | | | | | | |
| ## | $groups | |  | | | | | | | |
| ## | sqrt_Chlc | | groups | | | | | | | |
| ## | 15°C.1 0.5523801 | | a | | | | | | | |
| ## | 18°C.1 0.5484593 | | a | | | | | | | |
| ## | 15°C.2 0.5463147 | | a | | | | | | | |
| ## | 18°C.2 0.5383654 | | a | | | | | | | |
| ## | 18°C.3 0.5157069 | | a | | | | | | | |
| ## | 15°C.3 0.5094000 | | a | | | | | | | |
| ## |  | |  | | | | | | | |
| ## | attr(,"class") | |  | | | | | | | |
| ## | [1] "group" | |  | | | | | | | |
| tx <- **with**(AnalisisO2, **interaction**(Temperatura, Tiempo)) modelo <- **aov**(log_FX**~** tx, data=AnalisisO2)  out<-**HSD.test**(modelo, "tx", group=TRUE, alpha=0.05)  out | | | | | | | | | | |
| ## | $statistics | |  |  | |  | | | | |
| ## | MSerror Df | | Mean | CV | | MSD | | | | |

## 0.003347987 30 0.205664 28.13413 0.1016091 ##

## $parameters

## test name.t ntr StudentizedRange alpha

## Tukey tx 6 4.301464 0.05 ##

| ## | 11.22962 30 24.20873 13.84238 5.884686 |
| --- | --- |
| ## |  |
| ## | $parameters |
| ## | test name.t ntr StudentizedRange alpha |
| ## | Tukey tx 6 4.301464 0.05 |
| ## |  |

## $means

## log_FX std r se Min Max Q25

| ## | 15°C.1 | 0.2475639 | 0.04034389 | 6 | 0.02362198 | | 0.17679139 | 0.2988996 | 0.2404588 |
| --- | --- | --- | --- | --- | --- | --- | --- | --- | --- |
| ## | 15°C.2 | 0.2145954 | 0.04215362 | 6 | 0.02362198 | | 0.13832765 | 0.2625320 | 0.2076567 |
| ## | 15°C.3 | 0.1997789 | 0.05928736 | 6 | 0.02362198 | | 0.11456046 | 0.3003649 | 0.1889660 |
| ## | 18°C.1 | 0.2298815 | 0.07862233 | 6 | 0.02362198 | | 0.11052014 | 0.3013506 | 0.1791570 |
| ## | 18°C.2 | 0.1950184 | 0.06659284 | 6 | 0.02362198 | | 0.12258781 | 0.2764005 | 0.1458637 |
| ## | 18°C.3 | 0.1471460 | 0.05052024 | 6 | 0.02362198 | | 0.09671788 | 0.2212054 | 0.1051917 |
| ## |  | Q50 | Q75 |  |  | |  |  |  |
| ## | 15°C.1 | 0.2568309 | 0.2599796 | | | | | | |
| ## | 15°C.2 | 0.2278555 | 0.2295226 | | | | | | |
| ## | 15°C.3 | 0.1988179 | 0.2000272 | | | | | | |
| ## | 18°C.1 | 0.2600849 | 0.2863216 | | | | | | |
| ## | 18°C.2 | 0.1810647 | 0.2514132 | | | | | | |
| ## | 18°C.3 | 0.1428222 | 0.1757005 | | | | | | |
| ## |  |  |  | | | | | | |
| ## | $comparison | |  | | | | | | |
| ## | NULL | |  | | | | | | |
| ## |  | |  | | | | | | |
| ## | $groups | |  | | | | | | |
| ## | log_FX | | groups | | | | | | |
| ## | 15°C.1 0.2475639 | | a | | | | | | |
| ## | 18°C.1 0.2298815 | | a | | | | | | |
| ## | 15°C.2 0.2145954 | | a | | | | | | |
| ## | 15°C.3 0.1997789 | | a | | | | | | |
| ## | 18°C.2 0.1950184 | | a | | | | | | |
| ## | 18°C.3 0.1471460 | | a | | | | | | |
| ## |  | |  | | | | | | |
| ## | attr(,"class") | |  | | | | | | |
| ## | [1] "group" | |  | | | | | | |
| tx <- **with**(AnalisisO2, **interaction**(Temperatura, Tiempo)) modelo <- **aov**(sqrt_ROS**~** tx, data=AnalisisO2)  out<-**HSD.test**(modelo, "tx", group=TRUE, alpha=0.05) out | | | | | | | | | |
| ## | $statistics | |  |  | |  | | | |
| ## | MSerror Df | | Mean | CV | | MSD | | | |

| ## ## | $means | sqrt_ROS | std r | se | Min | Max | Q25 | Q50 |
| --- | --- | --- | --- | --- | --- | --- | --- | --- |
| ## | 15°C.1 21.29865 2.677681 6 1.368066 | | | | 18.08779 24.45229 18.93886 21.76672 | | | |
| ## | 15°C.2 21.32680 2.847461 6 1.368066 | | | | 17.16737 24.72329 19.67354 21.41198 | | | |
| ## | 15°C.3 24.06340 2.281102 6 1.368066 | | | | 21.54323 27.04431 22.04443 24.11471 | | | |
| ## | 18°C.1 26.12173 2.982302 6 1.368066 | | | | 23.04716 29.67154 23.39662 26.16543 | | | |
| ## | 18°C.2 25.35429 5.138983 6 1.368066 | | | | 15.48990 29.90833 25.10024 27.13702 | | | |
| ## | 18°C.3 27.08750 3.404856 6 1.368066 | | | | 22.19775 30.37653 24.59660 28.41269 | | | |
| ## | Q75 | | | |  | | | |

## 15°C.1 23.23329

## 15°C.2 23.46710

## 15°C.3 25.68550

## 18°C.1 28.44671

## 18°C.2 27.80836

## 18°C.3 29.45372 ##

## $comparison ## NULL

##

## $groups

## sqrt_ROS groups ## 18°C.3 27.08750 a

## 18°C.1 26.12173 a

## 18°C.2 25.35429 a

## 15°C.3 24.06340 a

## 15°C.2 21.32680 a

## 15°C.1 21.29865 a ##

## attr(,"class")

## [1] "group"

tx <- **with**(AnalisisO2, **interaction**(Temperatura, Tiempo)) modelo <- **aov**(sqrt_MDA**~** tx, data=AnalisisO2)

out<-**HSD.test**(modelo, "tx", group=TRUE, alpha=0.05) out

| ## | $statistics | |  | | | | | | |
| --- | --- | --- | --- | --- | --- | --- | --- | --- | --- |
| ## | MSerror Df | | Mean | CV | MSD | | | | |
| ## | 0.004480043 30 2.066474 3.239002 0.1175389 | | | | | | | | |
| ## |  | | | | | | | | |
| ## | $parameters | | | | | | | | |
| ## | test name.t ntr StudentizedRange alpha | | | | | | | | |
| ## | Tukey tx 6 4.301464 0.05 | | | | | | | | |
| ## |  | | | | | | | | |
| ## | $means |  |  |  | |  |  |  |  |
| ## |  | sqrt_MDA | std r | se | | Min | Max | Q25 | Q50 |
| ## | 15°C.1 | 2.007679 | 0.03352088 6 0.02732533 1.953430 | | | | 2.052537 1.995978 2.011578 | | |
| ## | 15°C.2 | 2.040012 | 0.06397502 6 0.02732533 1.957682 | | | | 2.139963 2.012195 2.023532 | | |
| ## | 15°C.3 | 2.090026 | 0.02898783 6 0.02732533 2.056511 | | | | 2.130608 2.073321 2.080685 | | |
| ## | 18°C.1 | 2.050439 | 0.04780687 6 0.02732533 2.008305 | | | | 2.137380 2.017915 2.040313 | | |
| ## | 18°C.2 | 2.077893 | 0.04042303 6 0.02732533 2.042223 | | | | 2.142895 2.045589 2.066031 | | |
| ## | 18°C.3 | 2.132794 | 0.13001535 6 0.02732533 2.042689 | | | | 2.385346 2.048708 2.094330 | | |
| ## |  | Q75 |  | | | |  | | |
| ## | 15°C.1 | 2.022523 | | | | | | | |
| ## | 15°C.2 | 2.071092 | | | | | | | |
| ## | 15°C.3 | 2.110770 | | | | | | | |
| ## | 18°C.1 | 2.059481 | | | | | | | |
| ## | 18°C.2 | 2.100060 | | | | | | | |
| ## | 18°C.3 | 2.133508 | | | | | | | |
| ## |  |  | | | | | | | |

## $comparison ## NULL

##

## $groups

## sqrt_MDA groups ## 18°C.3 2.132794 a

## 15°C.3 2.090026 ab

## 18°C.2 2.077893 ab

## 18°C.1 2.050439 ab

## 15°C.2 2.040012 ab

## 15°C.1 2.007679 b ##

## attr(,"class")

## [1] "group"

| ## | $statistics |  | | |
| --- | --- | --- | --- | --- |
| ## | MSerror Df | Mean | CV | MSD |

tx <- **with**(AnalisisO2, **interaction**(Temperatura, Tiempo)) modelo <- **aov**(sqrt_FI**~** tx, data=AnalisisO2)

out<-**HSD.test**(modelo, "tx", group=TRUE, alpha=0.05) out

| ## | 0.146419 30 2.927982 13.06863 0.6719536 | | | | | |  | | | |
| --- | --- | --- | --- | --- | --- | --- | --- | --- | --- | --- |
| ## |  | | | | | |  |  |  |  |
| ## | $parameters | | | | | |  |  |  |  |
| ## | test name.t ntr StudentizedRange alpha | | | | | |  |  |  |  |
| ## | Tukey tx 6 4.301464 0.05 | | | | | |  |  |  |  |
| ## |  | | | | | |  |  |  |  |
| ## | $means | | | | | |  |  |  |  |
| ## | sqrt_FI std r se | | | | | | Min | Max | Q25 | Q50 |
| ## | 15°C.1 | 2.413859 | 0.1642273 | 6 | 0.1562151 | 2.250258 | | 2.701741 | 2.306607 | 2.386059 |
| ## | 15°C.2 | 2.823276 | 0.2286166 | 6 | 0.1562151 | 2.540364 | | 3.078104 | 2.644946 | 2.825188 |
| ## | 15°C.3 | 3.136507 | 0.6311339 | 6 | 0.1562151 | 2.368014 | | 4.133805 | 2.818061 | 2.973252 |
| ## | 18°C.1 | 3.619699 | 0.2359029 | 6 | 0.1562151 | 3.377623 | | 4.060060 | 3.493974 | 3.589060 |
| ## | 18°C.2 | 2.841693 | 0.3364185 | 6 | 0.1562151 | 2.455701 | | 3.422056 | 2.635353 | 2.818578 |
| ## | 18°C.3 | 2.732860 | 0.4817883 | 6 | 0.1562151 | 2.250258 | | 3.619427 | 2.448974 | 2.654194 |
| ## |  | Q75 |  |  |  |  | |  |  |  |
| ## | 15°C.1 | 2.455701 | | | | | | | | |
| ## | 15°C.2 | 3.020756 | | | | | | | | |
| ## | 15°C.3 | 3.446604 | | | | | | | | |
| ## | 18°C.1 | 3.627349 | | | | | | | | |
| ## | 18°C.2 | 2.925367 | | | | | | | | |
| ## | 18°C.3 | 2.792439 | | | | | | | | |
| ## |  |  | | | | | | | | |

## $comparison ## NULL

##

## $groups

## sqrt_FI groups ## 18°C.1 3.619699 a

## 15°C.3 3.136507 ab

## 18°C.2 2.841693 bc

## 15°C.2 2.823276 bc

## 18°C.3 2.732860 bc

## 15°C.1 2.413859 c ##

## attr(,"class")

## [1] "group"

tx <- **with**(AnalisisO2, **interaction**(Temperatura, Tiempo)) modelo <- **aov**(sqrt_FS**~** tx, data=AnalisisO2)

out<-**HSD.test**(modelo, "tx", group=TRUE, alpha=0.05) out

| ## | $statistics | |  | | | | | | |
| --- | --- | --- | --- | --- | --- | --- | --- | --- | --- |
| ## | MSerror Df | | Mean | CV | | MSD | | | |
| ## | 0.06483547 30 3.758101 6.775445 0.4471435 | | | | | |  |  |  |
| ## |  | | | | | |  |  |  |
| ## | $parameters | | | | | |  |  |  |
| ## | test name.t ntr StudentizedRange alpha | | | | | |  |  |  |
| ## | Tukey tx 6 4.301464 0.05 | | | | | |  |  |  |
| ## |  | | | | | |  |  |  |
| ## | $means | | | | | |  |  |  |
| ## | sqrt_FS std r se Min | | | | | | Max | Q25 | Q50 |
| ## | 15°C.1 | 3.883190 | 0.3887067 | 6 | 0.1039515 | 3.448441 | 4.610608 | 3.758630 | 3.786682 |
| ## | 15°C.2 | 3.567825 | 0.2415275 | 6 | 0.1039515 | 3.212582 | 3.822420 | 3.435265 | 3.575201 |
| ## | 15°C.3 | 3.686948 | 0.2147047 | 6 | 0.1039515 | 3.465919 | 3.977361 | 3.515450 | 3.639018 |
| ## | 18°C.1 | 4.281104 | 0.2176983 | 6 | 0.1039515 | 4.067494 | 4.669211 | 4.138854 | 4.242019 |
| ## | 18°C.2 | 3.952505 | 0.1378176 | 6 | 0.1039515 | 3.814508 | 4.141107 | 3.853907 | 3.904207 |
| ## | 18°C.3 | 3.177036 | 0.2590367 | 6 | 0.1039515 | 2.822071 | 3.483310 | 2.989163 | 3.282329 |
| ## |  | Q75 |  |  |  |  |  |  |  |
| ## | 15°C.1 | 3.884758 | | | | | | | |
| ## | 15°C.2 | 3.768494 | | | | | | | |
| ## | 15°C.3 | 3.854337 | | | | | | | |
| ## | 18°C.1 | 4.331567 | | | | | | | |
| ## | 18°C.2 | 4.061450 | | | | | | | |
| ## | 18°C.3 | 3.296136 | | | | | | | |
| ## |  |  | | | | | | | |

## $comparison ## NULL

##

## $groups

## sqrt_FS groups ## 18°C.1 4.281104 a

## 18°C.2 3.952505 ab

## 15°C.1 3.883190 ab

## 15°C.3 3.686948 b

## 15°C.2 3.567825 bc

## 18°C.3 3.177036 c ##

## attr(,"class")

## [1] "group"

tx <- **with**(AnalisisO2, **interaction**(Temperatura, Tiempo)) modelo <- **aov**(sqrt_DPPH**~** tx, data=AnalisisO2)

out<-**HSD.test**(modelo, "tx", group=TRUE, alpha=0.05) out

## $statistics

## MSerror Df Mean CV MSD ## 0.02344767 30 2.832476 5.406095 0.2688998 ##

## $parameters

## test name.t ntr StudentizedRange alpha

## Tukey tx 6 4.301464 0.05 ##

| ## | 15°C.1 0.6968607 0.06443875 6 0.02429945 0.6326204 0.8056333 0.6526251 |
| --- | --- |
| ## | 15°C.2 0.7782794 0.06673646 6 0.02429945 0.7080935 0.8861255 0.7253062 |
| ## | 15°C.3 0.7082923 0.05904541 6 0.02429945 0.5916080 0.7483315 0.7110053 |
| ## | 18°C.1 0.7028354 0.05680571 6 0.02429945 0.6403124 0.8062258 0.6800661 |
| ## | 18°C.2 0.6936354 0.02537447 6 0.02429945 0.6633250 0.7348469 0.6763204 |
| ## | 18°C.3 0.6407720 0.07275611 6 0.02429945 0.5099020 0.7280110 0.6303934 |
| ## | Q50 Q75 |

## $means

## sqrt_DPPH std r se Min Max Q25 Q50

| ## | 15°C.1 | 2.983912 | 0.08361815 | 6 | 0.06251356 | 2.878915 | 3.064651 | 2.912240 | 3.007601 |
| --- | --- | --- | --- | --- | --- | --- | --- | --- | --- |
| ## | 15°C.2 | 2.858018 | 0.18492874 | 6 | 0.06251356 | 2.555624 | 3.045297 | 2.784914 | 2.873838 |
| ## | 15°C.3 | 2.799357 | 0.15742961 | 6 | 0.06251356 | 2.607703 | 3.038443 | 2.717398 | 2.755003 |
| ## | 18°C.1 | 2.978787 | 0.12893682 | 6 | 0.06251356 | 2.832211 | 3.107482 | 2.874170 | 2.984042 |
| ## | 18°C.2 | 2.837488 | 0.12014396 | 6 | 0.06251356 | 2.701775 | 2.988405 | 2.753918 | 2.810366 |
| ## | 18°C.3 | 2.537292 | 0.20893070 | 6 | 0.06251356 | 2.343259 | 2.918728 | 2.391786 | 2.516334 |
| ## |  | Q75 |  |  |  |  |  |  |  |
| ## | 15°C.1 | 3.050087 | | | | | | | |
| ## | 15°C.2 | 3.001638 | | | | | | | |
| ## | 15°C.3 | 2.890098 | | | | | | | |
| ## | 18°C.1 | 3.091557 | | | | | | | |
| ## | 18°C.2 | 2.936777 | | | | | | | |
| ## | 18°C.3 | 2.563204 | | | | | | | |
| ## |  |  | | | | | | | |

## $comparison ## NULL

##

## $groups

## sqrt_DPPH groups ## 15°C.1 2.983912 a

## 18°C.1 2.978787 a

## 15°C.2 2.858018 a

## 18°C.2 2.837488 a ## 15°C.3 2.799357 ab ## 18°C.3 2.537292 b ##

## attr(,"class")

## [1] "group"

tx <- **with**(AnalisisO2, **interaction**(Temperatura, Tiempo)) modelo <- **aov**(sqrt_aETR**~** tx, data=AnalisisO2)

out<-**HSD.test**(modelo, "tx", group=TRUE, alpha=0.05) out

| ## ## | $statistics  MSerror Df | | Mean | CV | MSD | | | |
| --- | --- | --- | --- | --- | --- | --- | --- | --- |
| ## | 0.003542779 30 0.7034459 8.461382 | | | | 0.1045232 | | | |
| ## |  | | | |  | | | |
| ## | $parameters | | | |  | | | |
| ## | test name.t ntr StudentizedRange | | | | alpha | | | |
| ## | Tukey tx 6 4.301464 | | | | 0.05 | | | |
| ## |  | | | |  | | | |
| ## | $means |  |  |  | |  |  |  |
| ## |  | sqrt_aETR | std r | se | | Min | Max | Q25 |

| ## | 15°C.1 0.6797745 | | | 0.7247834 | | | | | | |
| --- | --- | --- | --- | --- | --- | --- | --- | --- | --- | --- |
| ## | 15°C.2 0.7765570 | | | 0.8047300 | | | | | | |
| ## | 15°C.3 0.7314290 | | | 0.7399266 | | | | | | |
| ## | 18°C.1 0.6855655 | | | 0.7122241 | | | | | | |
| ## | 18°C.2 0.6964102 | | | 0.7000000 | | | | | | |
| ## | 18°C.3 0.6556995 | | | 0.6689465 | | | | | | |
| ## |  | | |  | | | | | | |
| ## | $comparison | | |  | | | | | | |
| ## | NULL | | |  | | | | | | |
| ## |  | | |  | | | | | | |
| ## | $groups | | |  | | | | | | |
| ## | sqrt_aETR | | | groups | | | | | | |
| ## | 15°C.2 0.7782794 | | | a | | | | | | |
| ## | 15°C.3 0.7082923 | | | ab | | | | | | |
| ## | 18°C.1 0.7028354 | | | ab | | | | | | |
| ## | 15°C.1 0.6968607 | | | ab | | | | | | |
| ## | 18°C.2 0.6936354 | | | ab | | | | | | |
| ## | 18°C.3 0.6407720 | | | b | | | | | | |
| ## |  | | |  | | | | | | |
| ## | attr(,"class") | | |  | | | | | | |
| ## | [1] "group" | | |  | | | | | | |
| tx <- **with**(AnalisisO2, **interaction**(Temperatura, Tiempo)) modelo <- **aov**(sqrt_ETR**~** tx, data=AnalisisO2)  out<-**HSD.test**(modelo, "tx", group=TRUE, alpha=0.05)  out | | | | | | | | | | |
| ## | $statistics | | |  |  |  | | | | |
| ## | MSerror Df | | | Mean | CV | MSD | | | | |
| ## | 0.4805796 30 | | 7.082778 9.787664 1.217372 | | | | | | | |
| ## |  | |  | | | | | | | |
| ## | $parameters | |  | | | | | | | |
| ## | test name.t | | ntr StudentizedRange alpha | | | | | | | |
| ## | Tukey tx | | 6 4.301464 0.05 | | | | | | | |
| ## |  | |  | | | | | | | |
| ## | $means |  |  | |  | |  |  |  |  |
| ## |  | sqrt_ETR | std r | | se | | Min | Max | Q25 | Q50 |
| ## | 15°C.1 | 6.355203 | 0.6588496 6 0.2830134 5.422167 7.009513 | | | | | | 5.880987 6.497323 | |
| ## | 15°C.2 | 6.561030 | 0.7834738 6 0.2830134 5.046498 7.315198 | | | | | | 6.598640 6.802089 | |
| ## | 15°C.3 | 8.214531 | 0.8240131 6 0.2830134 6.898707 9.166208 | | | | | | 7.858471 8.282653 | |
| ## | 18°C.1 | 7.044680 | 0.7162090 6 0.2830134 5.698524 7.819004 | | | | | | 7.068085 7.148924 | |
| ## | 18°C.2 | 7.489446 | 0.3942394 6 0.2830134 6.862616 7.971096 | | | | | | 7.344123 7.488392 | |
| ## | 18°C.3 | 6.831776 | 0.6987030 6 0.2830134 6.073064 7.801888 | | | | | | 6.332400 6.646385 | |
| ## |  | Q75 |  | | | | | |  | |
| ## | 15°C.1 | 6.896346 | | | | | | | | |
| ## | 15°C.2 | 6.852635 | | | | | | | | |
| ## | 15°C.3 | 8.775581 | | | | | | | | |
| ## | 18°C.1 | 7.345904 | | | | | | | | |
| ## | 18°C.2 | 7.744708 | | | | | | | | |
| ## | 18°C.3 | 7.357993 | | | | | | | | |
| ## |  |  | | | | | | | | |

## $comparison ## NULL

##

## $groups

## sqrt_ETR groups ## 15°C.3 8.214531 a

## 18°C.2 7.489446 ab

## 18°C.1 7.044680 ab

## 18°C.3 6.831776 b

## 15°C.2 6.561030 b

## 15°C.1 6.355203 b ##

## attr(,"class")

## [1] "group"

| ## | $statistics |  | | |
| --- | --- | --- | --- | --- |
| ## | MSerror Df | Mean | CV | MSD |

tx <- **with**(AnalisisO2, **interaction**(Temperatura, Tiempo)) modelo <- **aov**(sqrt_Ek**~** tx, data=AnalisisO2)

out<-**HSD.test**(modelo, "tx", group=TRUE, alpha=0.05) out

| ## | 4.052748 30 10.73675 18.75003 3.535212 | | | | | |  | | | |
| --- | --- | --- | --- | --- | --- | --- | --- | --- | --- | --- |
| ## |  | | | | | |  |  |  |  |
| ## | $parameters | | | | | |  |  |  |  |
| ## | test name.t ntr StudentizedRange alpha | | | | | |  |  |  |  |
| ## | Tukey tx 6 4.301464 0.05 | | | | | |  |  |  |  |
| ## |  | | | | | |  |  |  |  |
| ## | $means | | | | | |  |  |  |  |
| ## | sqrt_Ek std r se | | | | | | Min | Max | Q25 | Q50 |
| ## | 15°C.1 | 10.495841 | 1.150126 | 6 | 0.8218625 | 9.495266 | | 12.18554 | 9.718898 | 9.954075 |
| ## | 15°C.2 | 9.575798 | 1.906007 | 6 | 0.8218625 | 7.387635 | | 12.91674 | 8.706358 | 9.069109 |
| ## | 15°C.3 | 12.279857 | 1.902461 | 6 | 0.8218625 | 9.678832 | | 15.54372 | 11.660817 | 12.251256 |
| ## | 18°C.1 | 10.982252 | 1.720386 | 6 | 0.8218625 | 9.016630 | | 13.06625 | 9.684230 | 10.691896 |
| ## | 18°C.2 | 10.607652 | 2.320652 | 6 | 0.8218625 | 7.948029 | | 13.28748 | 8.758313 | 10.484644 |
| ## | 18°C.3 | 10.479084 | 2.719618 | 6 | 0.8218625 | 7.212728 | | 13.85248 | 8.611516 | 10.003544 |
| ## |  | Q75 |  |  |  |  | |  |  |  |
| ## | 15°C.1 | 11.29770 | | | | | | | | |
| ## | 15°C.2 | 10.08734 | | | | | | | | |
| ## | 15°C.3 | 12.43037 | | | | | | | | |
| ## | 18°C.1 | 12.48185 | | | | | | | | |
| ## | 18°C.2 | 12.56484 | | | | | | | | |
| ## | 18°C.3 | 12.74191 | | | | | | | | |
| ## |  |  | | | | | | | | |

## $comparison ## NULL

##

## $groups

## sqrt_Ek groups ## 15°C.3 12.279857 a

## 18°C.1 10.982252 a

## 18°C.2 10.607652 a

## 15°C.1 10.495841 a

## 18°C.3 10.479084 a

## 15°C.2 9.575798 a ##

## attr(,"class")

## [1] "group"

tx <- **with**(AnalisisO2, **interaction**(Temperatura, Tiempo)) modelo <- **aov**(sqrt_fvfm**~** tx, data=AnalisisO2)

out<-**HSD.test**(modelo, "tx", group=TRUE, alpha=0.05) out

| ## | $statistics | |  | | | | | |
| --- | --- | --- | --- | --- | --- | --- | --- | --- |
| ## | MSerror Df | | Mean | CV | | MSD | | |
| ## | 0.0009436776 30 0.8133186 3.777036 0.05394516 | | | | | |  |  |
| ## |  | | | | | |  |  |
| ## | $parameters | | | | | |  |  |
| ## | test name.t ntr StudentizedRange alpha | | | | | |  |  |
| ## | Tukey tx 6 4.301464 0.05 | | | | | |  |  |
| ## |  | | | | | |  |  |
| ## | $means | | | | | |  |  |
| ## | sqrt_fvfm std r se Min | | | | | | Max | Q25 |
| ## | 15°C.1 | 0.8450361 | 0.02393781 | 6 | 0.01254112 | 0.8089479 | 0.8715954 | 0.8306137 |
| ## | 15°C.2 | 0.8363921 | 0.03208865 | 6 | 0.01254112 | 0.7920267 | 0.8770175 | 0.8137322 |
| ## | 15°C.3 | 0.8256069 | 0.05039479 | 6 | 0.01254112 | 0.7403096 | 0.8770175 | 0.8039867 |
| ## | 18°C.1 | 0.8250706 | 0.02755399 | 6 | 0.01254112 | 0.7827233 | 0.8633729 | 0.8180798 |
| ## | 18°C.2 | 0.7803776 | 0.01594764 | 6 | 0.01254112 | 0.7533344 | 0.7949307 | 0.7737144 |
| ## | 18°C.3 | 0.7674281 | 0.02249848 | 6 | 0.01254112 | 0.7380609 | 0.7945366 | 0.7498928 |
| ## |  | Q50 | Q75 |  |  |  |  |  |
| ## | 15°C.1 | 0.8489007 | 0.8627405 | | | | | |
| ## | 15°C.2 | 0.8440074 | 0.8542417 | | | | | |
| ## | 15°C.3 | 0.8440074 | 0.8542417 | | | | | |
| ## | 18°C.1 | 0.8205334 | 0.8396326 | | | | | |
| ## | 18°C.2 | 0.7851285 | 0.7916573 | | | | | |
| ## | 18°C.3 | 0.7724810 | 0.7816047 | | | | | |
| ## |  |  |  | | | | | |
| ## | $comparison | |  | | | | | |
| ## | NULL | |  | | | | | |
| ## |  | |  | | | | | |
| ## | $groups | |  | | | | | |
| ## | sqrt_fvfm | | groups | | | | | |
| ## | 15°C.1 0.8450361 | | a | | | | | |
| ## | 15°C.2 0.8363921 | | a | | | | | |
| ## | 15°C.3 0.8256069 | | ab | | | | | |
| ## | 18°C.1 0.8250706 | | ab | | | | | |
| ## | 18°C.2 0.7803776 | | bc | | | | | |
| ## | 18°C.3 0.7674281 | | c | | | | | |
| ## |  | |  | | | | | |
| ## | attr(,"class") | |  | | | | | |
| ## | [1] "group" | |  | | | | | |
| tx <- **with**(AnalisisO2, **interaction**(Temperatura, Tiempo)) modelo <- **aov**(sqrt_NPQ**~** tx, data=AnalisisO2)  out<-**HSD.test**(modelo, "tx", group=TRUE, alpha=0.05)  out | | | | | | | | |
| ## | $statistics | |  |  | |  | | |
| ## | MSerror Df | | Mean | CV | | MSD | | |

## 0.02794721 30 1.572444 10.63149 0.2935688 ##

## $parameters

## test name.t ntr StudentizedRange alpha

## Tukey tx 6 4.301464 0.05 ##

## $means

## sqrt_NPQ std r se Min Max Q25 Q50

| ## | 15°C.1 | 1.331923 | 0.16456859 | 6 | 0.06824858 | 1.213526 | 1.557253 | 1.221735 | 1.234799 |
| --- | --- | --- | --- | --- | --- | --- | --- | --- | --- |
| ## | 15°C.2 | 1.384765 | 0.23924064 | 6 | 0.06824858 | 1.075409 | 1.797399 | 1.284727 | 1.360136 |
| ## | 15°C.3 | 1.576247 | 0.20266114 | 6 | 0.06824858 | 1.345774 | 1.817857 | 1.409840 | 1.581441 |
| ## | 18°C.1 | 1.651698 | 0.15361190 | 6 | 0.06824858 | 1.445328 | 1.815868 | 1.534054 | 1.687910 |
| ## | 18°C.2 | 1.682983 | 0.08336429 | 6 | 0.06824858 | 1.586388 | 1.784945 | 1.612990 | 1.688975 |
| ## | 18°C.3 | 1.807046 | 0.10838176 | 6 | 0.06824858 | 1.676409 | 1.951606 | 1.717915 | 1.839670 |
| ## |  | Q75 |  |  |  |  |  |  |  |
| ## | 15°C.1 | 1.459035 | | | | | | | |
| ## | 15°C.2 | 1.431974 | | | | | | | |
| ## | 15°C.3 | 1.729108 | | | | | | | |
| ## | 18°C.1 | 1.764781 | | | | | | | |
| ## | 18°C.2 | 1.742957 | | | | | | | |
| ## | 18°C.3 | 1.853112 | | | | | | | |
| ## |  |  | | | | | | | |

## $comparison ## NULL

##

## $groups

## sqrt_NPQ groups ## 18°C.3 1.807046 a

## 18°C.2 1.682983 a

## 18°C.1 1.651698 ab ## 15°C.3 1.576247 abc ## 15°C.2 1.384765 bc

## 15°C.1 1.331923 c ##

## attr(,"class")

## [1] "group"

position = **p**

*#GRaficas* **library**(ggplot2) **library**(ggpubr) **library**(dplyr)

**ggbarplot**(data=AnalisisO2, x="Tiempo", y="Chla", fill = "Temperatura", add = **c**("mean_se"), **scale_fill_brewer**(palette = "blue")

## Warning in pal_name(palette, type): Unknown palette blue

Temperatura 15°C 18°C

0.75

0.50

Chla

0.25

0.00

## 1 2 3

Tiempo

position = **p**

**ggbarplot**(data=AnalisisO2, x="Tiempo", y="Chlc", fill = "Temperatura", add = **c**("mean_se"), **scale_fill_brewer**(palette = "blue")

## Warning in pal_name(palette, type): Unknown palette blue

Temperatura 15°C 18°C

0.3

0.2

Chlc

0.1

0.0

1 2 3

Tiempo

**ggbarplot**(data=AnalisisO2, x="Tiempo", y="FX", fill = "Temperatura", add = **c**("mean_se"), position = **pos scale_fill_brewer**(palette = "blue")

## Warning in pal_name(palette, type): Unknown palette blue

Temperatura 15°C 18°C

1.5

1.0

FX

0.5

0.0

## 1 2 3

Tiempo

position = **po**

**ggbarplot**(data=AnalisisO2, x="Tiempo", y="ROS", fill = "Temperatura", add = **c**("mean_se"), **scale_fill_brewer**(palette = "blue")

## Warning in pal_name(palette, type): Unknown palette blue

Temperatura 15°C 18°C

800

600

400

ROS

200

0

1 2 3

Tiempo

position = **po**

**ggbarplot**(data=AnalisisO2, x="Tiempo", y="MDA", fill = "Temperatura", add = **c**("mean_se"), **scale_fill_brewer**(palette = "blue")

## Warning in pal_name(palette, type): Unknown palette blue

Temperatura 15°C 18°C

5

4

3

2

MDA

1

0

1 2 3

Tiempo

**ggbarplot**(data=AnalisisO2, x="Tiempo", y="FI", fill = "Temperatura", add = **c**("mean_se"), position = **pos scale_fill_brewer**(palette = "blue")

## Warning in pal_name(palette, type): Unknown palette blue

Temperatura 15°C 18°C

10

5

FI

0

1 2 3

Tiempo

**ggbarplot**(data=AnalisisO2, x="Tiempo", y="FS", fill = "Temperatura", add = **c**("mean_se"), position = **pos scale_fill_brewer**(palette = "blue")

## Warning in pal_name(palette, type): Unknown palette blue

Temperatura 15°C 18°C

20

15

10

FS

5

0

1 2 3

Tiempo

position = **p**

**ggbarplot**(data=AnalisisO2, x="Tiempo", y="DPPH", fill = "Temperatura", add = **c**("mean_se"), **scale_fill_brewer**(palette = "blue")

## Warning in pal_name(palette, type): Unknown palette blue

Temperatura 15°C 18°C

7.5

5.0

DPPH

2.5

0.0

## 1 2 3

Tiempo

position = **p**

**ggbarplot**(data=AnalisisO2, x="Tiempo", y="aETR", fill = "Temperatura", add = **c**("mean_se"), **scale_fill_brewer**(palette = "blue")

## Warning in pal_name(palette, type): Unknown palette blue

Temperatura 15°C 18°C

0.6

0.4

aETR

0.2

0.0

## 1 2 3

Tiempo

position = **po**

**ggbarplot**(data=AnalisisO2, x="Tiempo", y="ETR", fill = "Temperatura", add = **c**("mean_se"), **scale_fill_brewer**(palette = "blue")

## Warning in pal_name(palette, type): Unknown palette blue

Temperatura 15°C 18°C

60

40

ETR

20

0

1 2 3

Tiempo

**ggbarplot**(data=AnalisisO2, x="Tiempo", y="Ek", fill = "Temperatura", add = **c**("mean_se"), position = **pos scale_fill_brewer**(palette = "blue")

## Warning in pal_name(palette, type): Unknown palette blue

Temperatura 15°C 18°C

150

100

Ek

50

0

1 2 3

Tiempo

position = **p**

**ggbarplot**(data=AnalisisO2, x="Tiempo", y="fvfm", fill = "Temperatura", add = **c**("mean_se"), **scale_fill_brewer**(palette = "blue")

## Warning in pal_name(palette, type): Unknown palette blue

Temperatura 15°C 18°C

0.6

0.4

fvfm

0.2

0.0

## 1 2 3

Tiempo

position = **po**

**ggbarplot**(data=AnalisisO2, x="Tiempo", y="NPQ", fill = "Temperatura", add = **c**("mean_se"), **scale_fill_brewer**(palette = "blue")

## Warning in pal_name(palette, type): Unknown palette blue

Temperatura 15°C 18°C

3

2

NPQ

1

0

1 2 3

Tiempo

cor(x=AnalisisO2*FX, y* = *AnalisisO*2Chla)

with(AnalisisO2, plot(x=FX, y=Chla, pch=20, col=‘blue’, xlab=‘fucoxantina’, las=1, ylab=‘clorofila a’)) plot(x=AnalisisO2*FX, y* = *AnalisisO*2Chla)

str(AnalisisO2)
